# Global integrative analysis of whole-genome-based predictions of antimicrobial resistance trends in five major nosocomial pathogens

**DOI:** 10.1128/spectrum.00130-26

**Published:** 2026-08-19

**Authors:** Yen-Yi Liu, Li-Wen Huang, Mei-Fang Su, Bo-Ren Chen, Chih-Chieh Chen

**Affiliations:** 1 Department of Biology, National Changhua University of Education 34910, Changhua, Taiwan; 2 Institute of Medical Science and Technology, National Sun Yat-sen University 595943, Kaohsiung, Taiwan; 3 Department of Biomedical Science and Technology, National Sun Yat-sen University 34874, Kaohsiung, Taiwan; University of Saskatchewan, Saskatoon, Canada

**Keywords:** whole-genome-based prediction, antibiotic resistance fingerprints, nosocomial pathogens, antibiotic resistance

## Abstract

Global increases in antimicrobial resistance in key nosocomial pathogens, including *Acinetobacter baumannii*, *Enterococcus faecium*, *Klebsiella pneumoniae*, *Pseudomonas aeruginosa*, and *Staphylococcus aureus*, have severely compromised clinical efficacy and increased health-care expenditures. Although species-specific surveillance is common, large-scale analyses covering diverse geographic domains remain limited. To address this gap, we systematically analyzed more than 100,000 bacterial isolates from global databases. We leveraged whole-genome sequencing-based antibiotic resistance gene (ARG) identification from the curated 5NosoAE data set to characterize resistance fingerprints against 91 distinct antibiotics and disinfectants. We implemented a standardized high-specificity resistance fingerprint criterion based on strict ARG matching thresholds to generate standardized antibiotic resistance fingerprints to evaluate phenotypic distribution across the five aforementioned pathogens. Our findings revealed high-frequency resistance predominantly against frontline antibiotic classes such as aminoglycosides, folate pathway antagonists, and β-lactams. Specifically, we identified 209 resistance fingerprints that exhibited major co-occurrence patterns among the five nosocomial pathogens. This finding highlights the presence of shared resistance fingerprint patterns across species, suggesting that antimicrobial resistance surveillance may benefit from integrated multispecies comparative frameworks, in addition to isolated pathogen tracking. By establishing such a scalable, data-driven framework, this study provides a high-resolution framework for large-scale comparative surveillance of resistance fingerprint patterns. This framework can support evidence-based global antibiotic stewardship and the development of unified intervention strategies aimed at combating the expanding clinical resistome.

**IMPORTANCE:**

In this study, we established a standardized, scalable framework for multinational antimicrobial resistance analysis to address a major gap in current surveillance, namely, fragmented monitoring of individual pathogens. We observed extensive co-occurrence of resistance fingerprint patterns among five major nosocomial pathogens based on large-scale genomic surveillance data. These findings highlight the value of comparative multispecies surveillance frameworks for understanding large-scale resistance pattern distributions beyond isolated strain-level observations. Overall, our framework provides a high-resolution approach for comparative resistance fingerprint surveillance, supporting evidence-based antibiotic stewardship and future large-scale monitoring efforts based on publicly available genomic data sets.

## INTRODUCTION

### Increasing global threat of nosocomial antimicrobial resistance

Several antibiotics are primarily used in clinical practice to treat bacterial infections (1). However, the widespread and prolonged use of these agents has led to the development of multidrug-resistant bacterial strains in both hospital and community settings. A defining and deeply concerning characteristic of key global nosocomial pathogens, collectively known as the ESKAPE group, is their pronounced resistance to multiple antibiotic classes (2, 3). Of these pathogens, *Enterococcus faecium*, *Staphylococcus aureus*, *Klebsiella pneumoniae*, *Acinetobacter baumannii*, and *Pseudomonas aeruginosa* represent the most concerning threats to modern health care because their resistance profiles severely limit therapeutic options and are associated with increased mortality rates, prolonged hospital stays, and high health-care costs (3–11).

### Species-specific resistance profiles and clinical challenges

Several epidemiological studies have highlighted the remarkable adaptability of the aforementioned pathogens. For example, a previous study reported that the gram-negative opportunistic pathogen *A. baumannii* is highly resistant to carbapenems, which are often considered last-resort antibiotics (4). Other long-term surveillance studies, such as that conducted in Slovakia by Jalali et al. (5), have revealed an alarming increase in extensively drug-resistant *A. baumannii*, with more than 85% of the isolates exhibiting carbapenem resistance. Similarly, several studies have indicated that *K. pneumoniae* readily adapts to clinical environments by acquiring resistance and virulence plasmids. These studies have also revealed a global increase in the prevalence of carbapenem-resistant and hypervirulent strain fusion (11, 12), with high-risk clones often harboring extended-spectrum β-lactamase and carbapenemase genes (11, 13).

*E. faecium* is a gram-positive pathogen characterized by vancomycin resistance (3, 6, 7). This pathogen possesses a robust survival capability, forming biofilms that persist on hospital surfaces for extended periods (8–10). *S. aureus* also exhibits remarkable adaptability, with methicillin-resistant *S. aureus* being one of the most challenging clinical phenotypes (14, 15). Methicillin-resistant *S. aureus* strains often carry multiple resistance genes, including those for macrolides, aminoglycosides, tetracyclines, and fluoroquinolones, which complicates treatment (16, 17). *P. aeruginosa* utilizes its genomic plasticity and horizontal gene transfer (HGT) mechanisms to maintain high antimicrobial resistance (AMR) levels, particularly in intensive care units, where it is strongly associated with bacteremia (18).

### Current research gaps and the need for global connectivity analysis

Although extensive data are available on individual species, research has primarily focused on tracking the distribution and variation of AMR in specific bacterial strains or localized geographic regions (19, 20). This fragmented scope has limited the understanding of the global resistance landscape and hindered the potential for interspecies connectivity in resistance patterns across the nosocomial resistome. Specifically, limited research has compared the antibiotic resistance fingerprints, namely, the unique combinations of resistance traits, of the five aforementioned pathogens on a global scale. Understanding whether these fingerprints are shared across species boundaries is essential for identifying broad evolutionary trends and potential co-occurrence patterns.

### The 5NosoAE platform and the current study objectives

To address the aforementioned limitations, we previously launched the free online platform 5NosoAE (21). This platform is designed to help researchers analyze the antibiotic resistance fingerprints of the five predominant nosocomial pathogens of the ESKAPE group by mapping user-submitted genomic data onto a global predicted antibiogram. This functionality can help reduce antibiotic misuse and enhance genomic surveillance. In the present study, we analyzed the curated 5NosoAE data set, which comprises more than 100,000 isolate genomes collected between 2000 and 2021, to visualize global AMR trends and investigate the extent of resistance fingerprint pattern sharing and co-occurrence among five major nosocomial pathogens. By identifying these large-scale patterns, we provide novel insights into the ecological and evolutionary dynamics governing the nosocomial resistome, which may ultimately facilitate the development of more effective strategies for global AMR monitoring and control.

## MATERIALS AND METHODS

### Data source

This study analyzed the previously established 5NosoAE genomic data set, which comprises five major nosocomial pathogens: *A. baumannii*, *P. aeruginosa*, *K. pneumoniae*, *E. faecium*, and *S. aureus*. The data set includes genome assemblies, predicted antimicrobial resistance profiles, and associated isolate metadata. The construction of the 5NosoAE data set, including genomic data collection, quality control, genome assembly, antibiotic resistance gene (ARG) detection, and resistance profile generation, has been described previously (21) and is summarized in the Supplemental Materials and Methods.

The present study did not perform additional genome retrieval, *de novo* assembly, or ARG detection. All analyses were conducted using the curated 5NosoAE data set and its precomputed resistance profiles. Table S1 summarizes the cohort composition of the data set. Complete isolate-level metadata, including accession numbers and predicted resistance phenotypes, are publicly available through our GitHub repository (see Data Availability).

### Antibiotic resistance fingerprint classification

We developed a standardized phenotype-centered surveillance framework to investigate the connectivity of the nosocomial resistome. In this approach, a uniform set of 91 antibiotic markers was used as a common reference framework across all five pathogens, independent of their biological characteristics. Binary resistance profiles generated from these markers were defined as antibiotic resistance fingerprints. To identify identical resistance patterns across different species, Hamming distance was used to quantify differences between resistance fingerprints. Fingerprints differing by at least one antibiotic feature (Hamming distance ≥1) were assigned to different classes, whereas profiles with a Hamming distance of zero, indicating identical resistance signatures, were assigned to the same cluster. To validate the robustness of these shared signals and ensure that they were not artifacts of the strict *S*_EV_ = 100 threshold, sensitivity analyses were conducted using relaxed thresholds of *S*_EV_ ≥ 95 and *S*_EV_ ≥ 90. This step enabled us to evaluate the robustness of resistance fingerprint detection and downstream statistical analyses. By maintaining this consistent comparative scale, we treated intrinsic resistance as a stable genomic background. This approach ensured that the identified shared fingerprints and their statistical associations, such as odds ratios (ORs), reflected shared resistance fingerprint patterns across pathogens. Notably, we primarily focused on resistance fingerprints derived from ARG content rather than on the phylogenetic relationships between isolates. Therefore, we did not use any genome similarity clustering methods (e.g., whole-genome single-nucleotide polymorphism or core genome multilocus sequence typing) because these methods are based on core genome variations and may not accurately reflect variations in accessory resistance determinants.

### Statistical analysis

Fisher’s exact tests were applied to the consolidated co-occurrence matrices to evaluate the statistical significance of resistance pattern co-occurrence between species pairs. ORs accompanied by 95% confidence intervals were used to quantify the magnitude of these associations. Because of the high-throughput nature of testing required for examining 91 antibiotics, we controlled for the inflation of type I error rates by applying the Benjamini–Hochberg procedure to calculate the false discovery rate. Associations were considered significant only when the adjusted *P* value (false discovery rate) was <0.05. All genomic data processing, fingerprint clustering, and statistical modeling procedures were performed using R software (version 4.1.2; R Foundation for Statistical Computing, Vienna, Austria).

### Computational workflow and software resources

Table S3 summarizes the software tools and custom scripts used for the analytical procedures performed in this study, including resistance fingerprint classification, statistical analyses, and data visualization. Software tools used for the original construction of the 5NosoAE data set are described in the Supplemental Materials and Methods.

## RESULTS

### Population-level census of resistance determinants among nosocomial pathogens

In this study, 107,386 isolates were systematically analyzed, and a comprehensive landscape of 8,770 nonredundant predicted antibiotic resistance fingerprints was identified (Table 1). Surveillance data indicated that a majority of the global genomic data set, totaling 89,216 isolates (83.08%), harbored at least one genotypic resistance determinant; notably, 28,644 isolates (32.11%) exhibited resistance fingerprints characterized by interspecies co-occurrence. Although *K. pneumoniae* was responsible for the highest genomic data density (24,396 isolates and 5,565 fingerprints), its resistance landscape was highly specialized, with only 1.94% of its patterns crossing species boundaries. By contrast, *E. faecium* exhibited the highest degree of resistance fingerprint co-occurrence among the five nosocomial pathogens, with approximately 54.79% of its isolates and 16.35% of its resistance fingerprints also identified in other pathogen species. Moreover, major interspecies overlaps were recorded for *S. aureus* and *P. aeruginosa* at the isolate level, reaching 41.43% and 30.98%, respectively, with the sharing profile for *A. baumannii* remaining relatively constrained. Only 209 (2.38%) of the 8,770 nonredundant fingerprints were identified as interspecies shared patterns, suggesting that although critical overlaps exist, most of the nosocomial resistome remains structured by species-specific evolutionary constraints.

**TABLE 1:** Distribution of isolates and antibiogram patterns in the five major nosocomial pathogens

Species	No. of isolates			No. of observed antibiogram patterns	
	Total	With AMRs (%)	Co-occurrence (%)^b^	Total	Co-occurrence (%)
*A. baumannii*	10,782	9,840 (91.26)	1,223 (12.43)	960	72 (7.50)
*E. faecium*	16,235	15,168 (93.43)	8,311 (54.79)	532	87 (16.35)
*K. pneumoniae*	24,397	21,804 (89.37)	2,244 (10.29)	5,565	108 (1.94)
*P. aeruginosa*	13,957	6,726 (48.19)	2,084 (30.98)	725	95 (13.10)
*S. aureus*	42,016	35,678 (84.92)	14,782 (41.43)	1,240	99 (7.98)
Total (nr)^a^	107,387	89,216 (83.08)	28,644 (32.11)	9,022 (8,770)^a^	461 (209)^a^

^a^ nr refers to nonredundant fingerprint patterns remaining after the removal of duplicate patterns, not nonredundant isolates.

^b^ Co-occurrence percentages (%) are calculated using the number of AMR-positive isolates as the denominator.

### Geographic distribution of global genomic surveillance data

**Fig 1:**
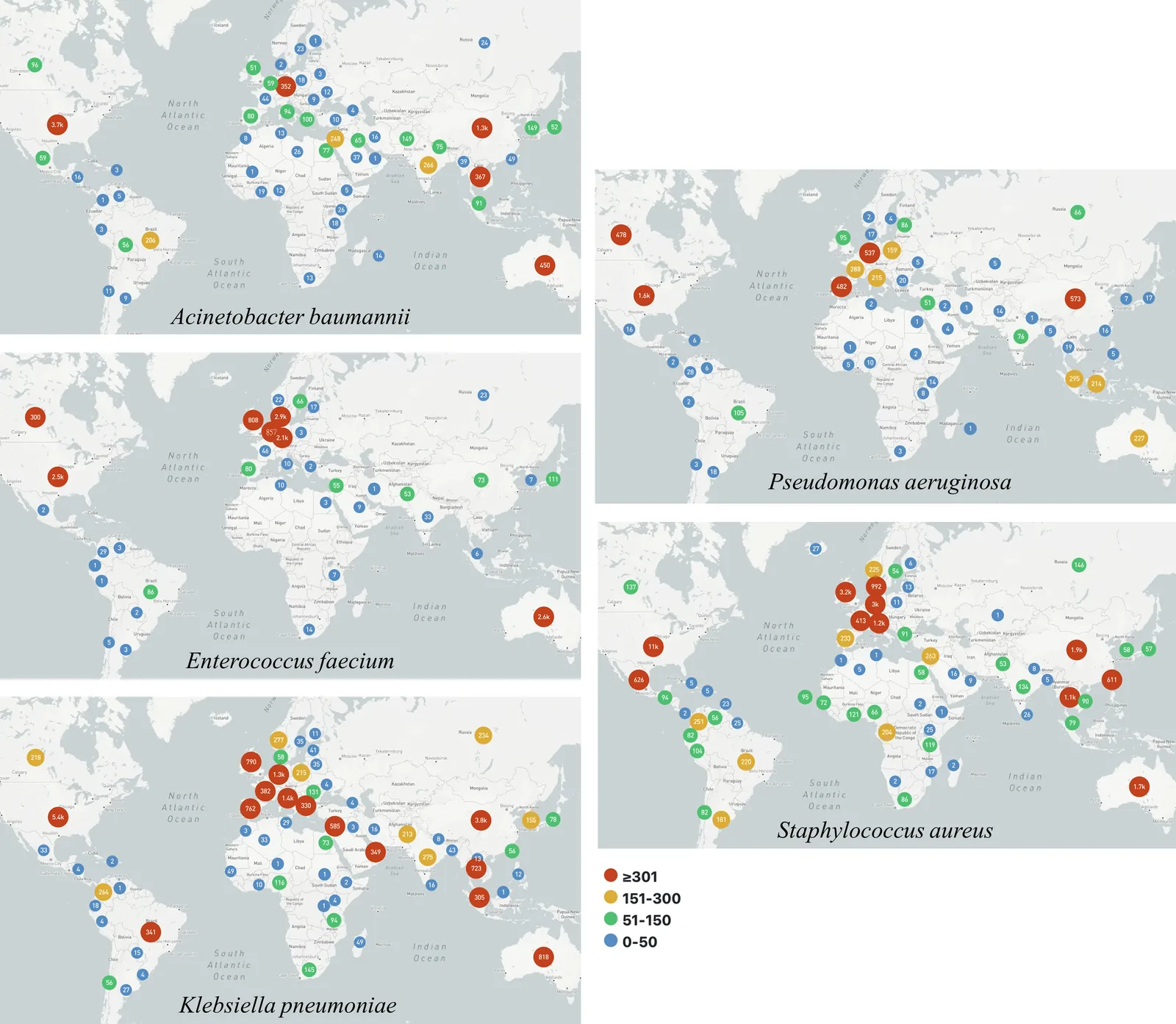
Global distribution maps of the five major nosocomial pathogens. This figure depict*s* global distribution maps of *A. baumannii*, *E. faecium*, *K. pneumoniae*, *P. aeruginosa*, and *S. aureus* based on data from the 5NosoAE platform. The colored circles represent the cumulative number of reported strains per region: red (≥301 isolates), yellow (151–300 isolates), green (51–150 isolates), and blue (0–50 isolates). Base map: Mapbox, OpenStreetMap contributors.

Figure 1 presents scatterplots illustrating the global geographic distribution of the five pathogens, where circle metrics correspond to the surveillance density recorded in specific regions. To facilitate a systematic assessment of regional disparities, global distribution was stratified into eight geographical domains: Asia, the Middle East, Europe, Africa, North America, South America, Australia, and Oceania. Our analysis revealed profound regional heterogeneities in resistance prevalence (Fig. S3). High-intensity surveillance signals were concentrated in Asia, particularly in China, India, and Southeast Asia, dominated by *K. pneumoniae* and *A. baumannii*. In the Middle East, surveillance records were predominantly associated with *A. baumannii*, *K. pneumoniae*, and *S. aureus*. In Europe, *A. baumannii* records were highly localized in western Germany, with other pathogens demonstrating a broad regional distribution. This study revealed a key finding from a public health perspective, that is, a major surveillance gap in Africa and Oceania, where sparse records constrained detailed statistical comparisons. Conversely, North America, specifically, the United States, maintained the most robust and comprehensive longitudinal data set for all five target species.

### Comparative analysis of public health burden by resistance class

**Fig 2:**
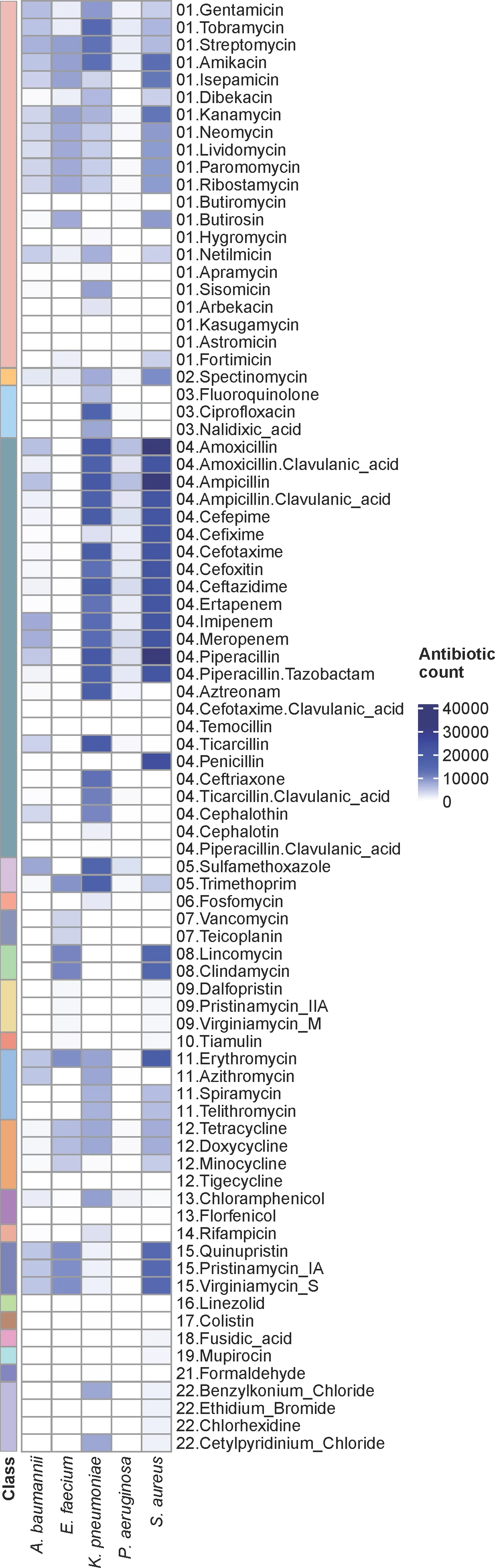
Heatmap of antibiotic counts associated with resistance genes in the five major nosocomial pathogens. This heatmap illustrates the number of antibiotics associated with resistance genes for each pathogen, with data obtained on more than 100,000 isolate records from the 5NosoAE platform. Color intensity represents cumulative antibiotic count, with darker shades indicating higher values.

**Fig 3:**
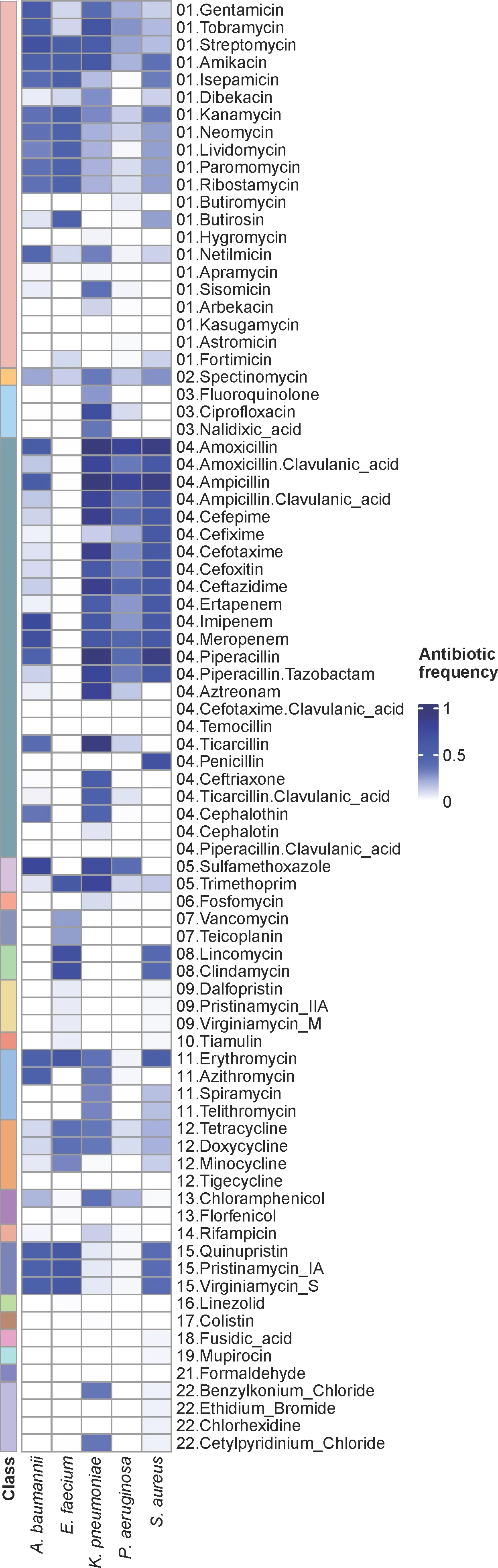
Heatmap of antibiotic frequencies associated with resistance genes in the five major nosocomial pathogens. The resistance profiles of the five pathogens were evaluated against 91 antibiotics. To account for differences in sample size among species, resistance data are expressed as frequencies rather than as cumulative counts. Color intensity represents the frequency of antibiotics associated with resistance genes, with darker shades indicating higher frequencies.

Absolute counts (Fig. 2) and relative frequency metrics (Fig. 3) were integrated to quantify the public health burden of resistance across 107,386 isolates. Surveillance data indicated that *A. baumannii* isolates exhibited high-frequency resistance to aminoglycosides (class 1), β-lactams (class 4), folate pathway antagonists (class 5), and streptogramin B (class 15). By contrast, *E. faecium* was characterized by high resistance against glycopeptides (class 7), lincosamides (class 8), and tetracyclines (class 12), but no resistance against β-lactams. *K. pneumoniae* exhibited the broadest multidrug resistance spectrum, with resistance against aminocyclitols (class 2), fluoroquinolones (class 3), macrolides (class 11), and quaternary ammonium compounds (class 22). *P. aeruginosa* exhibited a more specialized resistance pattern, with resistance primarily against β-lactams and folate pathway antagonists. *S. aureus* consistently exhibited high-frequency resistance to aminocyclitols, β-lactams, and lincosamides.

### Species-specific resistance fingerprint profiles

Fine-grained profiling provided comprehensive insights into the unique resistance signatures of individual pathogens in the global resistome (Fig. S4 through S8). Specifically, *A. baumannii* consistently exhibited high resistance against aminoglycosides and β-lactams but low resistance against phenicol (class 13; Fig. S4). *E. faecium* consistently demonstrated resistance against glycopeptides and tetracyclines (Fig. S5). *K. pneumoniae* exhibited a complex resistance pattern against fluoroquinolones and macrolides, along with exceptionally high resistance against quaternary ammonium compounds, reflecting environmental selective pressures (Fig. S6). *P. aeruginosa* exhibited high resistance against β-lactams but nearly no resistance against aminocyclitols or phenicol (Fig. S7). Finally, *S. aureus* demonstrated high resistance against lincosamides and streptogramin B and moderate resistance against aminoglycosides (Fig. S8).

### Dynamics of shared resistance fingerprints across pathogens

**Fig 4:**
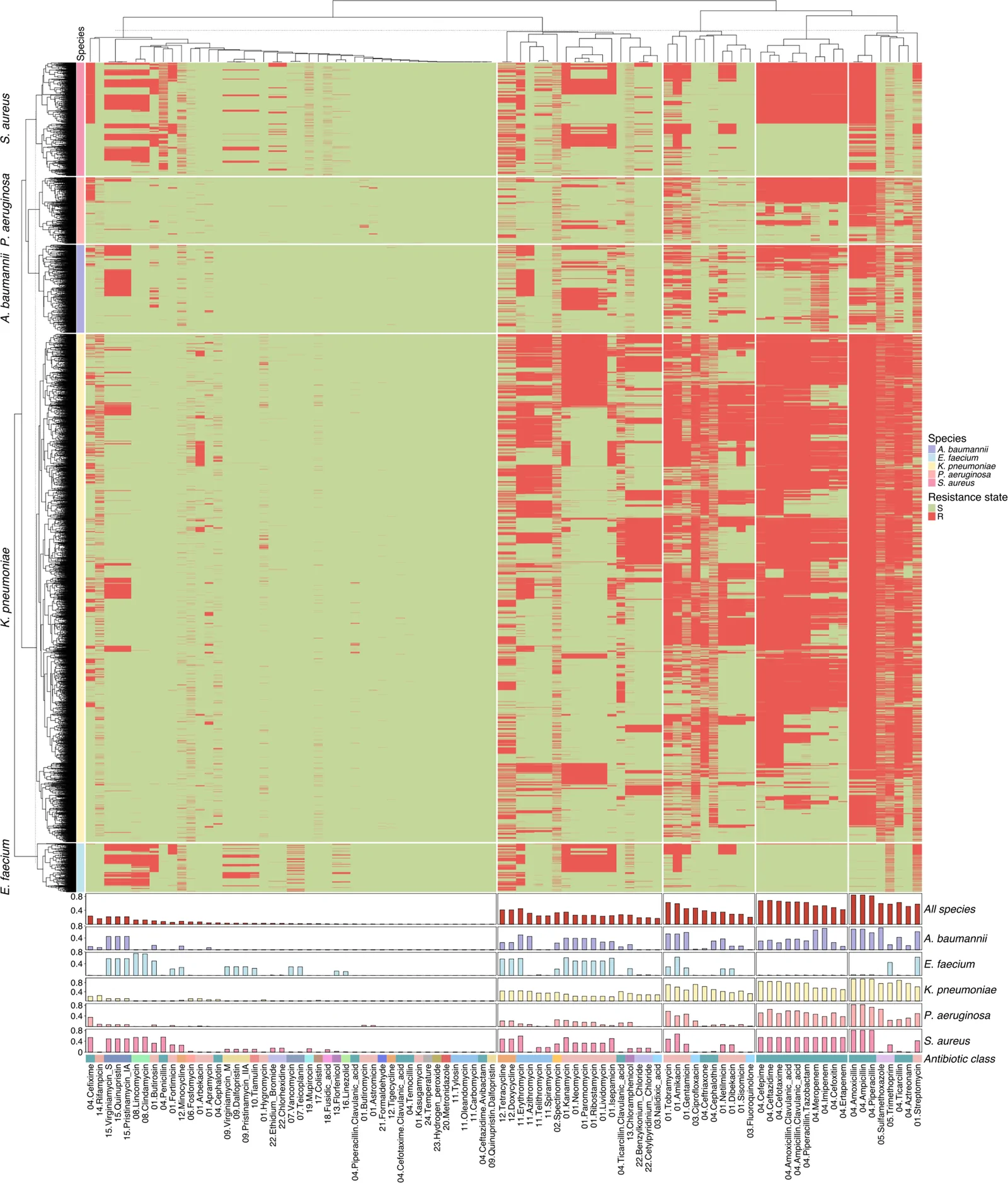
Heatmap and frequency distribution of antibiotic resistance fingerprints in the five major nosocomial pathogens. This heatmap illustrates the antibiotic resistance fingerprints of the five major nosocomial pathogens against 91 antibiotics, with duplicate resistance fingerprints removed for each species. Resistance data are displayed as a heatmap to visualize patterns across species and antibiotics. In addition, bar charts are used to summarize the frequency of resistance to each antibiotic for individual pathogens, as well as the overall frequency across all five pathogens.

To delineate the functional core of the shared nosocomial resistome, we subtyped various fingerprints to remove redundant patterns for each species. Figure 4 depicts an integrated heatmap of the apparent distribution of resistance classes in the shared global resistome. At a superficial level, antibiotic classes such as β-lactams, folate pathway antagonists, and aminoglycosides appear frequently across shared fingerprints. However, from a surveillance perspective, these high absolute counts may simply reflect the ubiquitous baseline prevalence of these classes in the clinical environment. Therefore, these observed frequencies represent a preliminary landscape that requires statistical correction to distinguish between common clinical presence and true interspecies enrichment.

### Interspecies connectivity and shared resistance risk assessment

**Fig 5:**
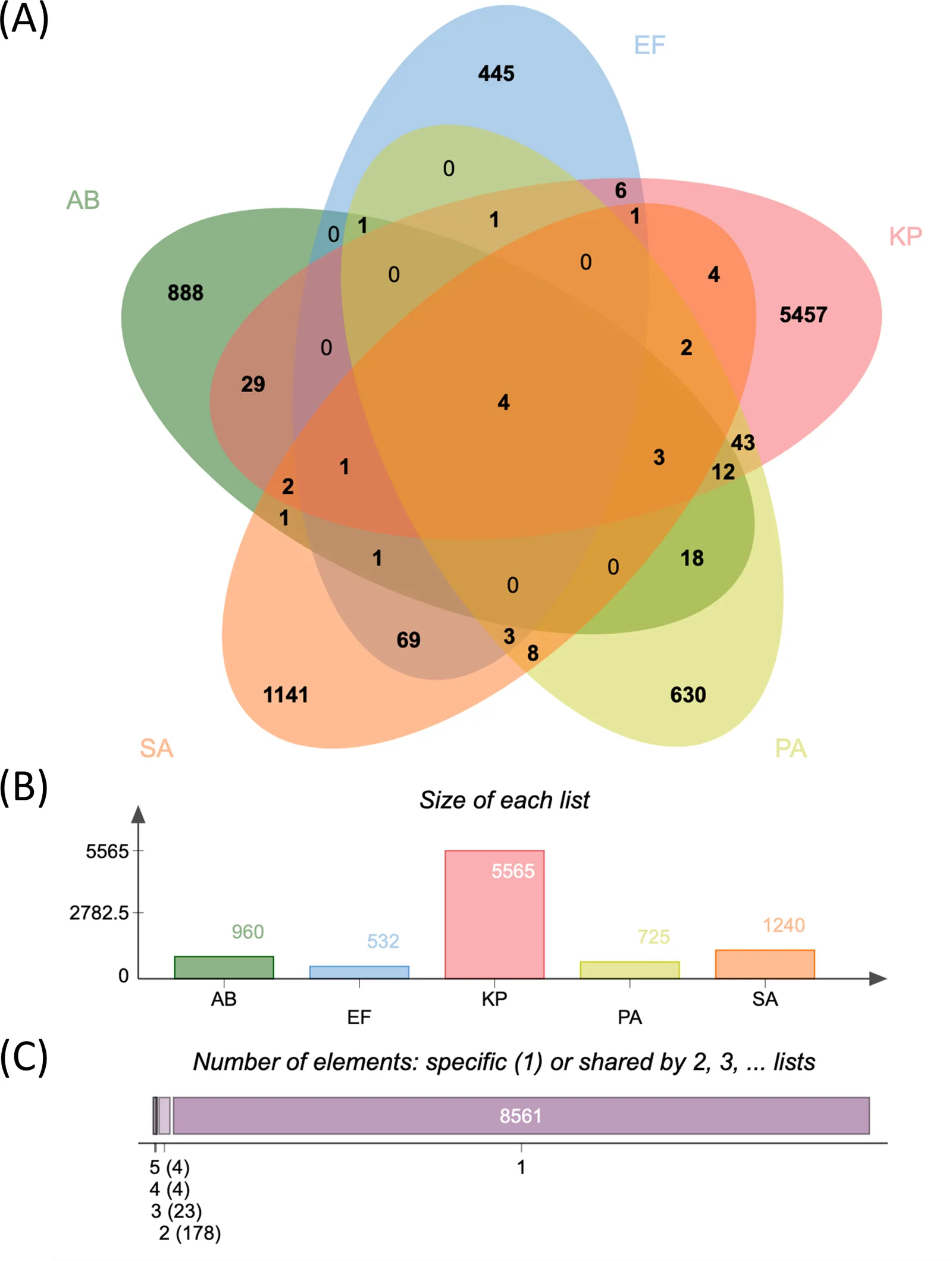
Comparison of the antibiotic resistance fingerprints of the five major nosocomial pathogens. (**A**) Venn diagram illustrating the overlap between 9,022 antibiotic resistance profiles from the five pathogens. (**B**) Bar chart depicting the number of antibiotic resistance profiles identified in each species. (**C**) Distribution of antibiotic resistance profiles identified in a single pathogen or shared among multiple nosocomial pathogens.

To investigate and quantify the interspecies co-occurrence of predicted antibiotic resistance fingerprints, we compared 8,770 nonredundant fingerprints from the five major nosocomial pathogens and illustrated their overlap with a Venn diagram (Fig. 5A). We identified the most frequent fingerprint presentation across two species in *E. faecium–S. aureus* (69 fingerprints), followed by *K. pneumoniae–P. aeruginosa* (43 fingerprints) and *K. pneumoniae–A. baumannii* (29 fingerprints). Among three-species combinations, the highest degree of sharing was that in *K. pneumoniae–A. baumannii–P. aeruginosa* (12 fingerprints). Patterns crossing four species boundaries were less common, with three fingerprints shared specifically by *S. aureus–K. pneumoniae–P. aeruginosa–A. baumannii* and one fingerprint shared by *S. aureus–K. pneumoniae–E. faecium–A. baumannii*. Notably, four predicted antibiotic resistance fingerprints were identified across all five species.

Figure 5B provides a comprehensive census of the predicted antibiotic resistance fingerprints identified in each species: *A. baumannii* (960), *E. faecium* (532), *K. pneumoniae* (5,565), *P. aeruginosa* (725), and *S. aureus* (1,240). Surveillance data revealed that although 8,561 fingerprints were species specific, 209 demonstrated interspecies co-occurrence in at least 2 species. Specifically, 178 fingerprints were present in 2 species, 23 fingerprints were present in 3 species, and 4 fingerprints were present in 4 species (Fig. 5C). Notably, four fingerprints were detected across the entire spectrum of all five pathogens, highlighting the presence of shared resistance fingerprint patterns across multiple species.

### Spatiotemporal distribution and persistence of shared fingerprints

**Fig 6:**
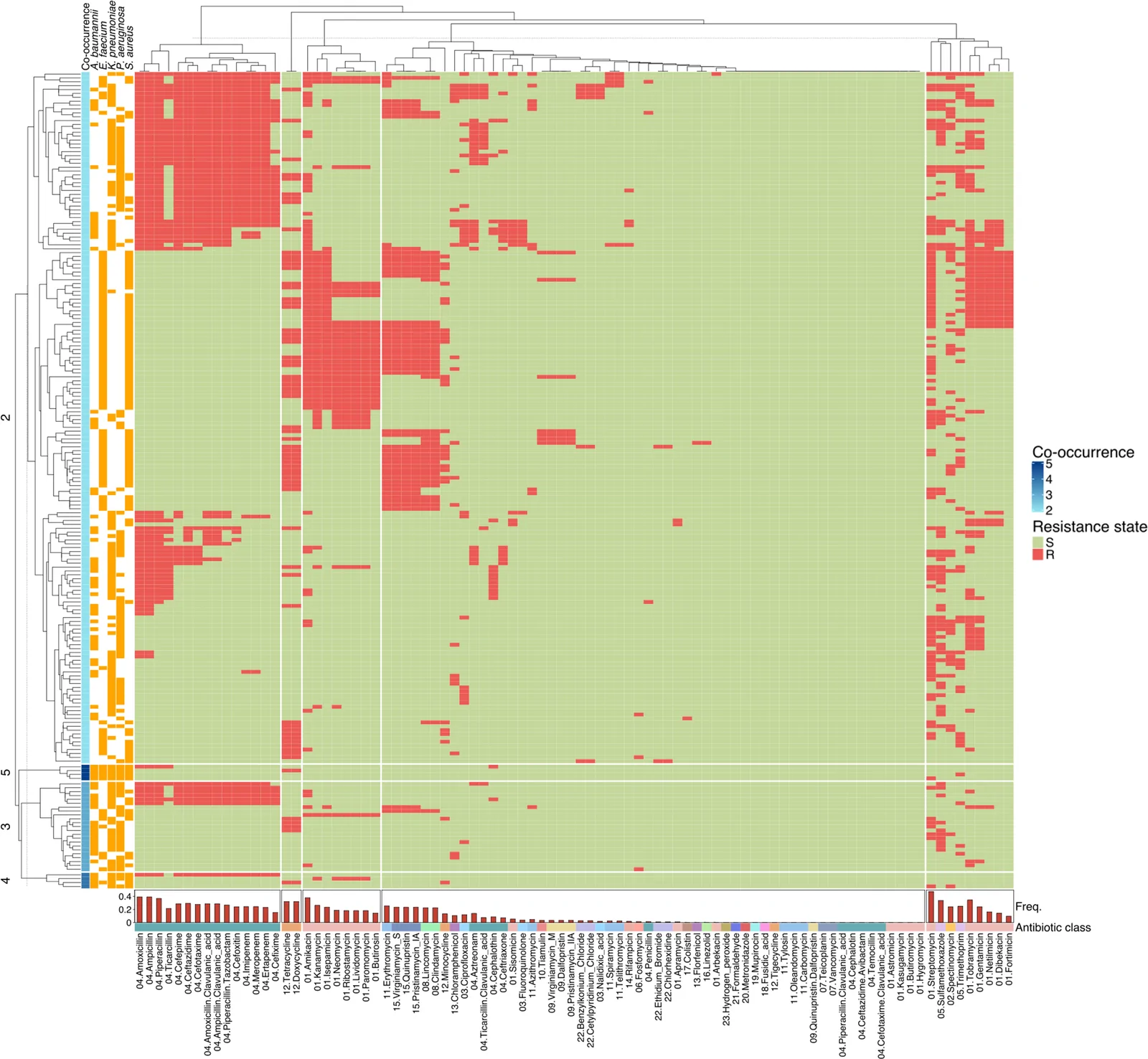
Heatmap of shared antibiotic resistance fingerprints clustered according to their occurrence among the five major nosocomial pathogens. A total of 209 shared antibiotic resistance fingerprints, defined as identical resistance profiles identified in two or more pathogens across 91 antibiotics, were identified. Each row represents an antibiotic resistance fingerprint. The columns correspond to the 91 antibiotics, and the orange annotations indicate the pathogens in which each fingerprint was detected. The accompanying bar chart summarizes the frequency of shared antibiotic resistance fingerprints across the 91 antibiotics.

**Fig 7:**
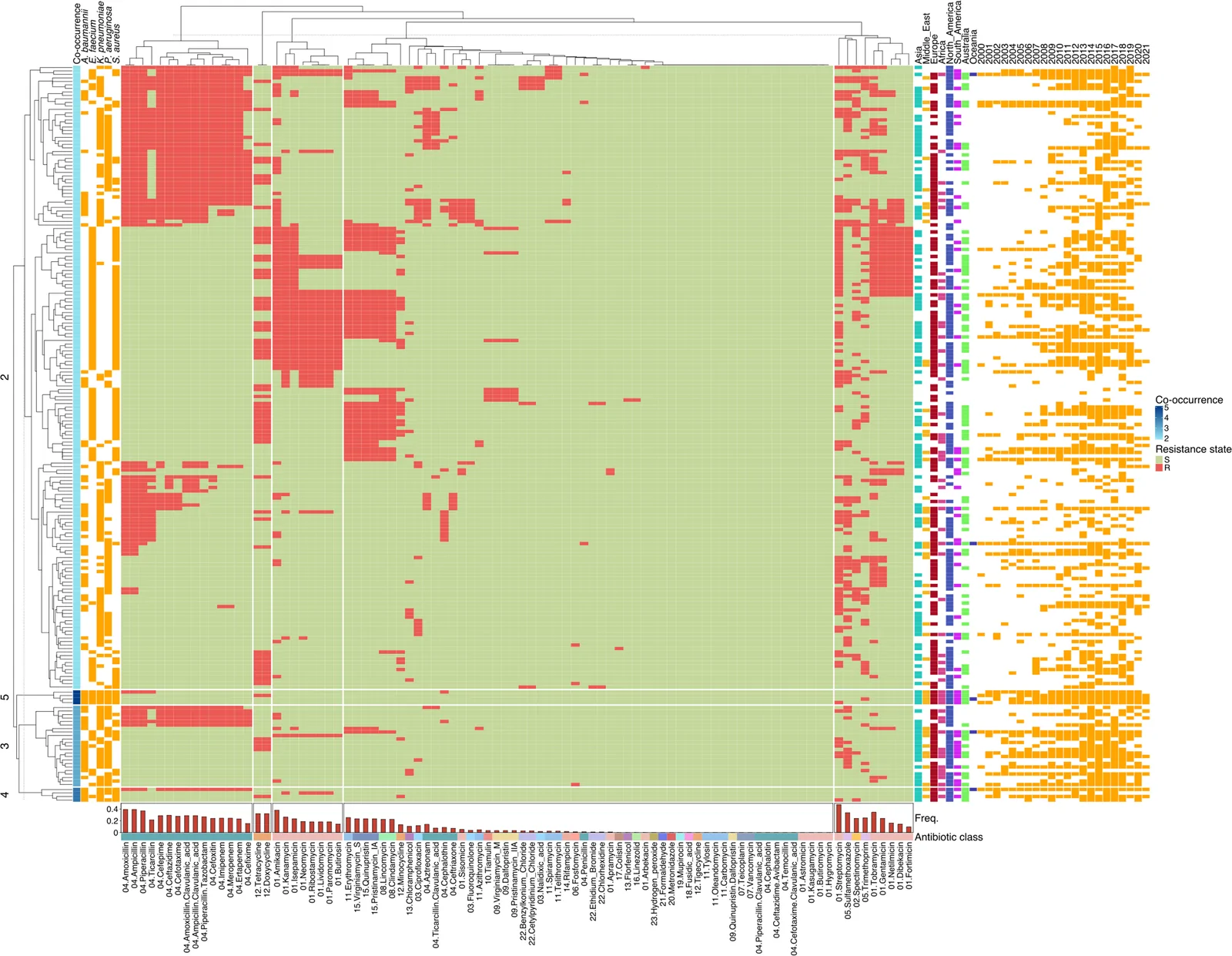
Temporal and regional distribution of shared antibiotic resistance fingerprints. The analysis includes temporal and geographical information associated with isolates carrying shared antibiotic resistance fingerprints. Data are derived from 209 antibiotic resistance fingerprints identified in two or more nosocomial pathogens and evaluated across 91 antibiotics and disinfectants. The bar charts summarize the temporal and geographic distributions of isolates carrying these shared resistance fingerprints. Colors in the temporal and regional panels indicate the presence of isolates with the corresponding resistance fingerprints in specific years or geographic regions.

In this study, we generated a heatmap with categorization by species to evaluate the distribution characteristics of the 209 shared fingerprints (Fig. 6). We incorporated regional and temporal metadata to assess global distribution patterns (Fig. 7). Although β-lactam resistance was high in *S. aureus* but nearly absent in *E. faecium*, we identified a critical shift. Specifically, as the β-lactam fingerprints in *S. aureus* declined over time, they were detected in *E. faecium* populations. Notably, the fingerprints identified across all five species demonstrated stable global persistence from 2000 to 2021, typically targeting antibiotic classes such as tetracyclines. In five species, shared fingerprints demonstrated temporal variations and reduced prevalence in South America, Africa, the Middle East, and Oceania. Systematic Fisher’s exact tests revealed that although the majority of traits exhibited statistical depletion (OR < 1, *P* < 0.05; Table S5), the absolute frequencies remained high. For example, tetracycline resistance was frequently observed but not statistically enriched (OR = 0.67, *P* = 0.007), indicating relative depletion in shared fingerprints. However, significant enrichment (OR > 1, *P* < 0.05) was observed for lincosamides (clindamycin, OR = 2.16; lincomycin, OR = 2.15), specific aminoglycosides (fortimicin, OR = 1.90; butirosin, OR = 1.63), and minocycline (OR = 1.69).

### Impact of surveillance thresholds (*S*_EV_) on signal detection resolution

To ensure the methodological rigor of our surveillance framework, we systematically validated the *S*_EV_ = 100 threshold against lower sensitivity levels (*S*_EV_ ≥ 95 and *S*_EV_ ≥ 90). Comparative analysis revealed that reducing the *S*_EV_ threshold from 100 to 95 or 90 substantially increased the merging of similar resistance profiles, thereby reducing fingerprint-level resolution (Table S6). Specifically, adopting a lower threshold resulted in an 85% reduction in the detection capability for unique interspecies co-occurrence signals, with the number of identified shared fingerprints decreasing from 209 to only 30.

Across the aforementioned thresholds, shifts in resistance frequency demonstrated an allelic collapse effect (Fig. S9). At *S*_EV_ = 95, the predicted resistance prevalence for certain high-burden species, such as *K. pneumoniae*, reached an artificial saturation point of 99.99%. From a public health monitoring perspective, this saturation masks fine-grained resistance profile distinctions and reduces the ability to distinguish unique resistance fingerprint patterns. Collectively, these results support the use of *S*_EV_ = 100 as a high-specificity criterion for identifying shared resistance fingerprints among different nosocomial pathogens.

### Detailed spatiotemporal patterns and persistence at the isolate level

Only 209 of the 8,770 predicted antibiotic resistance fingerprints identified through overlap analysis were classified as interspecies co-occurrence variants. However, a broader assessment of the surveillance database, which comprises more than 100,000 isolate entries, revealed that 28,644 records matched these interspecies co-occurrence fingerprints (Table 1). High-resolution analysis of these isolates revealed several distinct distribution patterns of shared resistance fingerprints. First, the distribution density of a given co-occurrence fingerprint substantially varied across species; for example, fingerprint number 103 was identified in 22 *A. baumannii* isolates but was localized to only one *K. pneumoniae* isolate. Second, isolates from different species harboring identical co-occurrence fingerprints were not consistently restricted to the same geographical region, indicating geographically distributed occurrence across regions. Third, more than half of the co-occurrence fingerprints involved isolates from different species collected from the same geographical region; however, the sampling dates often exhibited major temporal disparities. Although some isolates overlapped in time, others were collected during distinct periods, indicating long-term persistence of shared resistance fingerprint patterns in public genomic records from regional health-care environments rather than transient, localized outbreaks.

### Global distribution of shared resistance fingerprints

**Fig 8:**
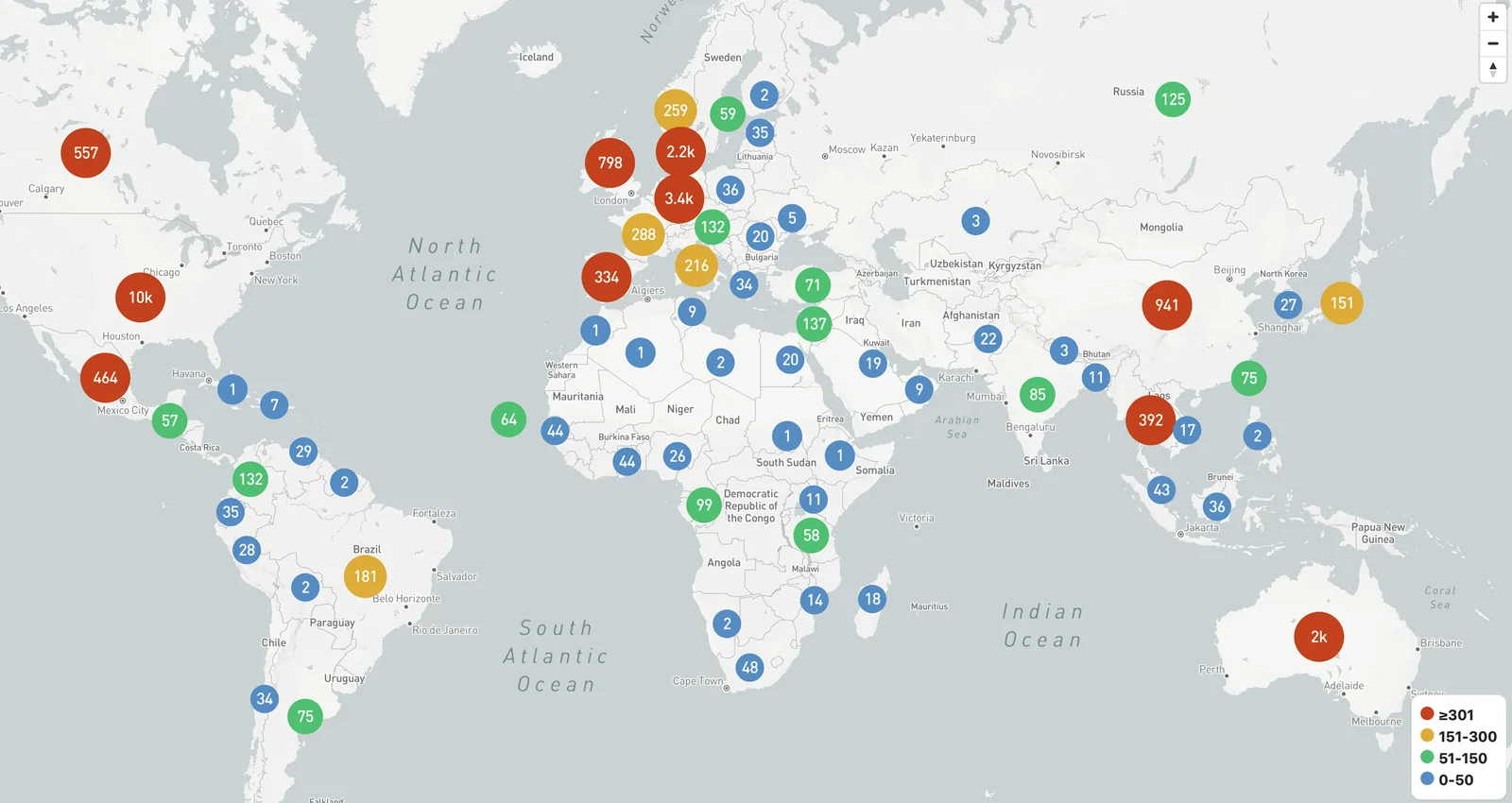
Global distribution of shared antibiotic resistance fingerprint patterns among the five major nosocomial pathogens. This map illustrates the global distribution of 24,669 isolates associated with 209 shared antibiotic resistance fingerprints identified in previous analyses. Of the 28,644 isolates exhibiting shared resistance fingerprint patterns (Table 1), 24,669 had associated geographic metadata and were therefore included in this map. Regions with high frequencies of shared resistance fingerprint patterns generally coincided with regions showing high representation of the five major nosocomial pathogens in public genomic databases, including China, Europe, North America, and Australia. Circles of different colors represent the cumulative number of strains reported in each region: red (≥301 isolates), yellow (151–300 isolates), green (51–150 isolates), and blue (0–50 isolates). Base map: Mapbox, OpenStreetMap contributors.

To identify key areas for intervention, we mapped the distribution of the 209 shared fingerprints (Fig. 8). Spatial analysis revealed high frequencies of shared resistance fingerprint patterns in China, Europe, North America, and Australia. These regions overlap with areas characterized by high representation of the five major pathogens in public genomic databases, likely reflecting regional surveillance density and data availability.

## DISCUSSION

### Co-occurrence of resistance fingerprints in the nosocomial resistome

In the present study, we selected *A. baumannii*, *E. faecium*, *K. pneumoniae*, *P. aeruginosa*, and *S. aureus* as our target microorganisms because of their status as major global nosocomial pathogens. These species consistently exhibit high AMR trends that severely limit therapeutic options (3, 22). We observed partial overlaps in predicted resistance fingerprints, suggesting interspecies co-occurrence. Although these similarities indicate phenotype-level co-occurrence of resistance patterns, they do not constitute evidence of gene transmission and may instead reflect independent adaptation to similar selective pressures. Notably, gram-negative pathogens (*A. baumannii*, *K. pneumoniae*, and *P. aeruginosa*) exhibited resistance against aminoglycosides, β-lactams, and folate pathway antagonists, whereas gram-positive pathogens (*E. faecium* and *S. aureus*) exhibited distinct resistance profiles associated with streptogramins and lincosamides. Despite their divergent evolutionary pathways, the high prevalence of β-lactam resistance remains a unifying clinical challenge across all five pathogens.

### Global distribution and sampling limitations

Previous AMR research has often been limited by localized geographic scopes. In this study, we established a large-scale data set containing more than 100,000 entries to facilitate the visualization of global resistance patterns. This geographic concentration likely reflects the combined influence of surveillance density, public database representation, and regional resistance prevalence patterns. Notably, major gaps remain in data coverage for Africa and parts of Oceania, and public repositories remain inherently biased toward North America and Europe. Consequently, absolute resistance frequencies should be interpreted as representative of current genomic databases rather than of precise global epidemiological prevalence. To address this limitation, we utilized ORs to focus on the relative association of traits, which helped neutralize the impact of potential clonal and geographic sampling inflation. Because ORs evaluate the relative association of resistance patterns between shared and nonshared groups, this approach is less sensitive to uniform clonal expansion in a single species compared with absolute frequency measures. Notably, public genomic data sets may involve clonal overrepresentation, which may influence species-specific frequency estimates. In this study, we primarily focused on identical resistance fingerprints (*S*_EV_ = 100) observed across species. The finding of these specific patterns being detected in multiple regions—even in the presence of sampling disparities—suggests that they represent stable components of the currently available genomic data set rather than artifacts driven only by regional sampling density.

### Statistical inference and mechanistic distinctions

Our systematic Fisher’s exact tests revealed that although the majority of resistance traits exhibited statistical depletion (OR < 1), specific antibiotic classes, such as lincosamides and certain aminoglycosides, were substantially enriched (OR > 1). This finding suggests that although the majority of shared fingerprints are constrained by genomic barriers, certain antibiotic classes were more frequently observed among shared resistance fingerprint patterns. Notably, the shared fingerprints identified in this study reflect phenotype-level co-occurrence based on trait co-occurrence. These findings do not necessarily indicate identical shared ARGs across species, nor do they provide direct evidence of HGT events. In the present study, we primarily focused on the functional outcome of the resistome rather than on the specific genetic vehicles or individual gene transfer mechanisms. Sensitivity analyses further revealed that antibiotic-level enrichment signals were dependent on the ARG identity threshold. When relaxed thresholds (*S*_EV_ ≥ 95 and *S*_EV_ ≥ 90) were implemented, reductions in pattern diversity and saturation effects limited the interpretability of the enrichment analyses. At *S*_EV_ = 100, tetracycline resistance was not enriched but instead demonstrated relative depletion, suggesting that shared resistance patterns are constrained by species-specific genomic contexts rather than broadly shared across different nosocomial pathogens. Notably, the resistance fingerprints analyzed in this study represent combinations of predicted phenotypic resistance traits derived from ARG detection, rather than from the direct identification of specific shared ARGs. Therefore, the observed patterns reflect the co-occurrence of resistance phenotypes rather than gene-level transmission events.

### Methodological robustness and future directions

To ensure high-resolution characterization, we applied a strict *S*_EV_ = 100 threshold. Although we adopted a conservative approach, our sensitivity analysis confirmed that lower thresholds resulted in “allelic collapse,” which obscured the fine-grained signals necessary for drawing statistical inferences. Overall, our data indicate that interspecies resistance is constrained by a complex interplay of ecological, geographical, and temporal factors. Although the genomic and experimental evidence provided in this study remains insufficient to confirm HGT, our findings provide a robust framework for future research. Likewise, the analytical framework used in this study does not support direct inference of transmission dynamics, dissemination routes, or epidemiological circulation patterns. Further targeted investigations are warranted to clarify whether these fingerprints result from direct transmission or parallel evolutionary adaptation. Such investigations can aid in the development of more effective interventions aimed at addressing shared resistance patterns among major pathogens.

### Conclusion

In summary, this study provides a large-scale genomic surveillance overview of AMR trends in five major nosocomial pathogens, with this achieved through a novel antibiotic resistance fingerprinting system. Our findings indicate that although resistance fingerprints are frequently shared across species, these overlaps are statistically constrained by species-specific factors, as indicated by widespread depletion (OR < 1), rather than being driven by generalized enrichment. These shared patterns represent phenotype-level co-occurrence and do not constitute direct evidence of HGT. Although certain geographical sampling biases exist, our findings highlight the presence of geographically distributed shared resistance fingerprint patterns observed in currently available public genomic data sets. Because publicly available genomic data sets remain affected by geographic sampling imbalance and limited phylogenetic resolution, future studies integrating larger and more geographically balanced data sets, together with phylogenetic and experimental approaches, will be necessary to further clarify the ecological and evolutionary drivers underlying the sharing of resistance fingerprint patterns among different nosocomial pathogens.

## Data Availability

The complete isolate-level metadata for all analyzed genomes, including accession numbers and predicted antibiotic resistance phenotypes, are available in a publicly accessible GitHub repository: https://github.com/cenesis/5NosoAE-antibiogram-analysis.

## SUPPLEMENTAL MATERIAL

Supplemental materials spectrum.00130-26-s0001.pdf Supplemental methods, Tables S1 to S6, and Figures S1 to S9.

ASM does not own the copyrights to Supplemental Material that may be linked to, or accessed through, an article. The authors have granted ASM a non-exclusive, world-wide license to publish the Supplemental Material files. Please contact the corresponding author directly for reuse.
