# Supplementary material for: Global integrative analysis of whole-genome-based predictions of antimicrobial resistance trends in five major nosocomial pathogens
Source: Microbiol Spectr. 2026 Aug 19;14(10):e00130-26. doi: 10.1128/spectrum.00130-26 (PMC13689369; doi:10.1128/spectrum.00130-26)
Supplement: Supplementary file 1 [file spectrum.00130-26-s0001.pdf]

SUPPLEMENTARY MATERIALS AND METHODS

Overview of the 5NosoAE Dataset

The following section summarizes the original construction and curation of the 5NosoAE dataset, which has been described previously (1). These procedures were performed during the development of the 5NosoAE platform and were not part of the analytical workflow of the present study**.** The analyses reported in this manuscript relied exclusively on the curated 5NosoAE dataset and its precomputed resistance profiles.

The 5NosoAE dataset comprises genome assemblies and associated metadata for five major nosocomial pathogens: *A. baumannii*, *P. aeruginosa*, *K. pneumoniae*, *E. faecium*, and *S. aureus*. The dataset was originally constructed using genome assemblies and associated sequencing records retrieved from the NCBI Assembly and Sequence Read Archive (SRA) databases.

To ensure the integrity and consistency of the dataset, a predefined set of inclusion criteria was applied during the original data collection and preprocessing. All publicly available isolates meeting these criteria were retained without removing potential clonal duplicates to preserve the genomic diversity represented in public repositories. For genomes obtained from the NCBI Assembly database, only the most recent ("latest version") assemblies were included, whereas records flagged as anomalous or lacking verified taxonomy were excluded.

For isolates with raw sequencing data available in the NCBI SRA database, only Illumina paired-end sequencing datasets with sequencing depths between 80× and 500× were included. Raw SRA files were downloaded using the SRA Toolkit (prefetch and fasterq-dump, version 2.11.1) (2). Sequencing reads were quality-filtered using fastp (version 1.1.0) (3) with automatic adapter detection and default quality-filtering settings, followed by de novo genome assembly using SKESA (version 2.2) (4) with default parameters. Assembly quality was subsequently assessed using QUAST (version 5.3.0) (5) based on assembly statistics including contig number, N50, and total assembly length before inclusion in the 5NosoAE dataset.

Of the 107,386 isolates included in the 5NosoAE dataset, 38,353 isolates lacked pre-existing genome assemblies in the NCBI Assembly database and were therefore processed through the raw-read retrieval, quality filtering, and de novo assembly pipeline described above. The remaining isolates were retrieved directly from the NCBI Assembly database as

pre-existing genome assemblies and were not subjected to de novo assembly or the assembly-quality assessment described below.

Assembly Quality of the Original 5NosoAE Genome Assemblies

The following assembly-quality metrics pertain to the original construction of the 5NosoAE dataset and were not newly generated or independently revalidated as part of the present study. Assembly quality statistics for the de novo genomes are summarized in **Table S4**. Overall, the assembly quality of the dataset was high across the five major nosocomial pathogens. The mean contig numbers ranged from 72.5 to 172.1, while the mean N50 values ranged from 48.9 kb to 205.1 kb. In addition, the total assembly lengths and GC contents were consistent with the known genomic characteristics of the corresponding bacterial species. The distributions of assembly quality metrics were generally compact and highly concentrated within species, indicating overall consistency and stability of the assembled genomes (**Figure S1**). A total of 37,993 of the 38,353 genomes (99.06%) satisfied the predefined assembly quality criteria (N50 ≥ 10 kb and contig number ≤ 1000) (**Figure S2**), indicating that the vast majority of genomes were suitable for downstream antimicrobial resistance profiling analyses.

Construction of Predicted Antibiotic Resistance Fingerprints

Genomic sequences in the 5NosoAE dataset were converted into resistance profiles by identifying antibiotic resistance genes (ARGs) and mapping them to their established clinical phenotypes. ARG detection was performed using ResFinder (version 4.2.3) in combination with resfinder_db (version 2.0.0) (6). The analyses were conducted using the parameters `--min_cov 0.4 -t 0.4 --acquired` to maximize the distribution range of sequence evidence values (S_{EV}) for downstream resistance profile comparisons.

The standardized surveillance profile comprised 91 antibiotics representing 24 pharmacological classes (**Table S2**). Resistance evidence for each isolate was quantified using the sequence evidence value (S_{EV}), which integrates sequence identity (SI) and alignment coverage (AC) relative to reference resistance determinants. S_{EV} was calculated as follows:

$$S_{EV} = (SI + AC)/2 \qquad (1)$$

For antibiotics associated with multiple detected ARGs, the highest S_{EV} was retained as the representative resistance value for that antibiotic. This metric provides a consistent, high-resolution measure of resistance evidence across the entire 5NosoAE dataset.

ARG detection and resistance profile generation were performed during the original construction of the 5NosoAE dataset, whereas the present study used the precomputed resistance profiles for binary fingerprint classification and downstream analyses. Specifically, resistance profiles were converted into binary resistance fingerprints using a strict "perfect match" criterion for interspecies comparison analyses. An isolate was classified as resistant (assigned a value of "1" in the binary antibiogram) only if its S_{EV} reached 100; all other values were classified as nonresistant ("0").

SUPPLEMENTARY TABLES

Table S1. Name of nosocomial pathogens and genome data distribution by source of database.

Nosocomial pathogens	Assembly[a]	SRA[b]	Total
Acinetobacter baumannii	9208	1574	10782
Enterococcus faecium	11729	4506	16235
Klebsiella pneumoniae	14766	9631	24397
Pseudomonas aeruginosa	8331	5626	13957
Staphylococcus aureus	25000	17016	42016
Total	69034	38353	107387

[a] Assembly filter criteria: latest version file, exclude anomalous, and taxonomy check.
[b] SRA filter criteria: genome, paired end, Illumina, and sequencing depth between 80× and 500×.

Table S2. List of 91 clinically relevant antibiotic resistance genes identified in this study, grouped into 24 functional categories based on resistance mechanisms or target antibiotic classes.

Class & subclass	Antibiotic name	Treatment line	Description
1. Aminoglycoside			
1-1	Gentamicin	2nd-3rd	Used for severe Gram-negative infections; nephrotoxic and ototoxic.
1-2	Tobramycin	2nd-3rd	More effective against *Pseudomonas aeruginosa*; inhaled form for cystic fibrosis.
1-3	Streptomycin	2nd	Used for tuberculosis and plague; resistance limits use.
1-4	Amikacin	2nd-3rd	Effective against multidrug-resistant Gram-negative bacteria.
1-5	Isepamicin	2nd-3rd	Similar to Amikacin, effective against resistant bacteria.
1-6	Dibekacin	2nd-3rd	Used against resistant Gram-negative infections.
1-7	Kanamycin	2nd	Mainly used for tuberculosis.
1-8	Neomycin	2nd	Used orally for bowel decontamination; also topical.
1-9	Lividomycin	2nd	Similar to Gentamicin.
1-10	Paromomycin	2nd	Used orally for amoebiasis and intestinal infections.
1-11	Ribostamycin	2nd	Similar to Gentamicin.
1-12	Butiromycin	2nd	Similar to Gentamicin.
1-13	Butirosin	2nd	Similar to Gentamicin.
1-14	Hygromycin	2nd	Primarily used in veterinary medicine.
1-15	Netilmicin	2nd-3rd	Similar to Amikacin; effective against resistant bacteria.
1-16	Apramycin	2nd	Used in veterinary medicine.
1-17	Sisomicin	2nd	Similar to Gentamicin.
1-18	Arbekacin	3rd	Effective against MRSA, but rarely used in clinical practice.
1-19	Kasugamycin	-	Used in agriculture, not for clinical use.
1-20	Astromicin	2nd-3rd	Similar to Amikacin.
1-21	Fortimicin	2nd-3rd	Similar to Amikacin.
2. Aminocyclitol			
2-1	Spectinomycin	2nd	Used for gonorrhea treatment in penicillin-allergic patients.
3. Fluoroquinolone			
3-1	Fluoroquinolone	2nd-3rd	Broad-spectrum antibiotics inhibiting DNA gyrase.
3-2	Ciprofloxacin	2nd	Used for UTIs, respiratory, and GI infections.
3-3	Nalidixic acid	1st	First-generation quinolone, mostly replaced by newer fluoroquinolones.
4. Beta-lactam			
4-1	Amoxicillin	1st	Broad-spectrum penicillin, often used for respiratory infections.
4-2	Amoxicillin Clavulanic acid	1st	Beta-lactamase inhibitor combination for resistant bacteria.
4-3	Ampicillin	1st	Used for a variety of infections, including *Listeria*.
4-4	Ampicillin Clavulanic acid	1st	Broad-spectrum beta-lactamase inhibitor combination.
4-5	Cefepime	3rd-4th	4th-generation cephalosporin; broad-spectrum, including *Pseudomonas*.
4-6	Cefixime	3rd	Oral 3rd-generation cephalosporin, used for respiratory and UTI infections.
4-7	Cefotaxime	3rd	Broad-spectrum cephalosporin for severe infections.
4-8	Cefoxitin	2nd	Used for anaerobic and intra-abdominal infections.
4-9	Ceftazidime	3rd	Strong activity against *Pseudomonas aeruginosa*.
4-10	Ertapenem	2nd-3rd	Carbapenem used for broad-spectrum infections but lacks *Pseudomonas* coverage.

4-11	Imipenem	2nd	Carbapenem with broad-spectrum activity; often combined with cilastatin.
4-12	Meropenem	2nd	Carbapenem with fewer CNS side effects than imipenem.
4-13	Piperacillin	2nd	Broad-spectrum penicillin for serious Gram-negative infections.
4-14	Piperacillin Tazobactam	2nd	Extended-spectrum beta-lactamase inhibitor combination.
4-15	Aztreonam	2nd	Monobactam effective against Gram-negative bacteria, including *Pseudomonas*.
4-16	Clavulanic acid	-	Beta-lactamase inhibitor, used in combination with other antibiotics.
4-17	Temocillin	2nd	Beta-lactam with activity against *Pseudomonas* and resistant bacteria.
4-18	Ticarcillin	2nd	Penicillin used for Gram-negative infections, often combined with clavulanic acid.
4-19	Ceftazidime Avibactam	3rd	Combination to combat resistant Gram-negative bacteria.
4-20	Penicillin	1st	Foundational antibiotic for Gram-positive infections.
4-21	Ceftriaxone	3rd	Broad-spectrum cephalosporin used for severe infections.
4-22	Ticarcillin Clavulanic acid	2nd	Combines extended-spectrum penicillin with a beta-lactamase inhibitor.
4-23	Cephalothin	1st	First-generation cephalosporin used for surgical prophylaxis.
4-24	Cephalotin	1st	First-generation cephalosporin used for Gram-positive infections.
4-25	Piperacillin Clavulanic acid	2nd	Extended-spectrum penicillin with beta-lactamase inhibition.
5. Folate Pathway Antagonist			
5-1	Sulfamethoxazole	2nd	Inhibits folic acid synthesis; often combined with trimethoprim.
5-2	Trimethoprim	2nd	Used for UTIs; often in combination with sulfamethoxazole.
6. Fosfomycin			
6-1	Fosfomycin	1st	Used for uncomplicated UTIs.
7. Glycopeptide			
7-1	Vancomycin	2nd	Used for MRSA and severe Gram-positive infections.
7-2	Teicoplanin	2nd	Used for similar infections as Vancomycin; fewer side effects.
8. Lincosamide			
8-1	Lincomycin	2nd	Used for Gram-positive infections, including anaerobes.
8-2	Clindamycin	2nd	Active against anaerobic and Gram-positive infections.
9. Streptogramin A			
9-1	Dalfopristin	3rd	Used with quinupristin for resistant Gram-positive infections.
9-2	Pristinamycin IIA	3rd	Similar to Dalfopristin, used for resistant infections.
9-3	Virginiamycin M	3rd	Used in agriculture, not commonly in clinical settings.
9-4	Quinupristin Dalfopristin	3rd	Combines both agents for resistant Gram-positive bacteria.
10. Pleuromutilin			
10-1	Tiamulin	-	Mainly used in veterinary medicine.
11. Macrolide			
11-1	Carbomycin	2nd	Used for Gram-positive infections, including *Streptococcus*.
11-2	Erythromycin	1st	Used for respiratory infections, alternative for penicillin allergy.
11-3	Azithromycin	2nd	Broad-spectrum macrolide; used for respiratory and STDs.

11-4	Oleandomycin	2nd	Used for Gram-positive infections.
11-5	Spiramycin	2nd	Used for respiratory infections and toxoplasmosis.
11-6	Tylosin	2nd	Used for animal infections, especially in poultry.
11-7	Telithromycin	2nd	Used for respiratory infections, particularly *Streptococcus* pneumoniae.
12. Tetracycline			
12-1	Tetracycline	1st	Broad-spectrum antibiotic.
12-2	Doxycycline	1st	Used for respiratory, skin, and tick-borne infections.
12-3	Minocycline	1st	Similar to doxycycline, used for acne and resistant infections.
12-4	Tigecycline	2nd	Broad-spectrum, used for multidrug-resistant infections.
13. Phenicol			
13-1	Chloramphenicol	3rd	Rarely used due to toxicity; used for meningitis and typhoid fever.
13-2	Florfenicol	3rd	Veterinary use for respiratory infections in animals.
14. Rifamycin			
14-1	Rifampicin	1st	Key drug for tuberculosis treatment.
15. Streptogramin B			
15-1	Quinupristin	3rd	Part of the combination for resistant Gram-positive infections.
15-2	Pristinamycin IA	3rd	Part of the combination for resistant infections.
15-3	Virginiamycin S	3rd	Used for animal infections.
16. Oxazolidinone			
16-1	Linezolid	3rd	Used for MRSA and VRE infections.
17. Polymyxin			
17-1	Colistin	3rd	Used for multidrug-resistant Gram-negative infections.
18. Steroid Antibacterial			
18-1	Fusidic acid	2nd	Used for skin infections caused by *Staphylococcus*.
19. Pseudomonic Acid			
19-1	Mupirocin	2nd	Used topically for skin infections, particularly MRSA.
20. Nitroimidazole			
20-1	Metronidazole	1st	Effective against anaerobic bacteria and protozoa.
21. Aldehyde			
21-1	Formaldehyde	-	Used for disinfection and sterilization.
22. Quaternary Ammonium Compounds (QAC)			
22-1	Benzalkonium Chloride	-	Used as a disinfectant and antiseptic.
22-2	Ethidium Bromide	-	Used in molecular biology for DNA staining.
22-3	Chlorhexidine	-	Used as a disinfectant and antiseptic.
22-4	Cetylpyridinium Chloride	-	Used in mouthwashes and antiseptics.
23. Peroxide			
23-1	Hydrogen Peroxide	-	Used for disinfection and wound cleaning.
24. Heat			
24-1	Temperature	-	Used in sterilization processes (e.g., autoclaving).

Table S3. Software tools and custom scripts used in the analytical workflow of the present study.

Workflow step	Software/Tool	Version	Description
Statistical analysis	Bash / Perl	v5.1.16 / v5.34.0	Calculation of resistance pattern frequency and co-occurrence statistics
Data visualization and clustering	R	v4.1.2	Heatmaps, frequency plots, clustering analysis, and statistical visualization

Table S4. Summary statistics of de novo genome assemblies for five major nosocomial pathogens.

Species	Number of isolates	Mean of contigs	Mean of N50 (kb)	Mean of total length (kb)	Mean of GC (%)
AB	1,574	132.44	132.80	3,984.83	39.08
EF	4,506	172.12	48.95	2,911.76	37.90
KP	9,631	108.55	205.09	5,577.66	57.15
PA	5,626	145.83	192.83	6,610.25	66.19
SA	17,016	72.55	173.97	2,786.22	32.72

These metrics pertain to the original construction of the 5NosoAE dataset and were not newly generated as part of the present study.

Table S5. Enrichment and depletion analysis of antibiotic resistance traits in shared versus non-shared fingerprints using Fisher's exact test.

Antibiotic classes	OR	P_value	FDR
OR > 1			
08.Clindamycin	2.15851418	2.10E-05	4.98E-05
08.Lincomycin	2.14912607	2.20E-05	4.99E-05
01.Fortimicin	1.90279348	0.01177176	0.02077369
12.Minocycline	1.68849911	0.01626745	0.02614411
01.Butirosin	1.59468172	0.02374013	0.03748441
10.Tiamulin	1.18584643	0.67047968	0.84990382
15.Quinupristin	1.11406915	0.49723971	0.64857353
15.Pristinamycin_IA	1.11406915	0.49723971	0.64857353
15.Virginiamycin_S	1.11406915	0.49723971	0.64857353
09.Dalfopristin	1.0507587	0.8414088	0.99640516
09.Pristinamycin_IIA	1.0507587	0.8414088	0.99640516
09.Virginiamycin_M	1.0507587	0.8414088	0.99640516
OR < 1			
04.Amoxicillin	0.1109942	6.91E-51	3.11E-49
04.Ampicillin	0.1109942	6.91E-51	3.11E-49
04.Piperacillin	0.11818795	7.38E-49	2.21E-47
04.Ticarcillin	0.15620268	1.25E-34	2.82E-33
04.Ceftazidime	0.17958666	2.11E-32	3.79E-31
04.Cefepime	0.18409734	1.82E-31	2.73E-30
04.Cefotaxime	0.18659864	9.98E-31	1.28E-29
04.Aztreonam	0.14628228	1.78E-30	2.00E-29
03.Ciprofloxacin	0.14990591	2.55E-27	2.55E-26
04.Ceftriaxone	0.11477572	6.24E-27	5.45E-26
04.Piperacillin.Tazobactam	0.20733148	6.66E-27	5.45E-26
04.Amoxicillin.Clavulanic_acid	0.2151938	3.60E-26	2.50E-25
04.Ampicillin.Clavulanic_acid	0.2151938	3.60E-26	2.50E-25
05.Trimethoprim	0.23012393	5.03E-23	3.23E-22
11.Azithromycin	0.10598367	4.35E-22	2.61E-21
04.Cephalothin	0.17133926	6.24E-19	3.51E-18
11.Telithromycin	0.07515304	7.90E-19	4.18E-18
11.Spiramycin	0.07552405	1.29E-18	6.47E-18
01.Sisomicin	0.13352452	5.05E-18	2.39E-17
04.Meropenem	0.27535532	9.46E-18	4.18E-17
04.Imipenem	0.27560816	9.76E-18	4.18E-17

01.Tobramycin	0.30615306	1.47E-16	6.03E-16
05.Sulfamethoxazole	0.32677806	5.40E-15	2.11E-14
04.Ticarcillin.Clavulanic_acid	0.21386591	5.24E-13	1.95E-12
04.Cefoxitin	0.33217463	5.42E-13	1.95E-12
22.Benzylkonium_Chloride	0.12420167	2.47E-12	8.24E-12
22.Cetylpyridinium_Chloride	0.12420167	2.47E-12	8.24E-12
03.Nalidixic_acid	0.09519233	5.51E-12	1.77E-11
03.Fluoroquinolone	0.15576528	8.89E-12	2.76E-11
14.Rifampicin	0.09819007	1.37E-11	4.12E-11
01.Gentamicin	0.37281931	1.35E-10	3.91E-10
01.Amikacin	0.4090317	3.18E-10	8.93E-10
01.Netilmicin	0.35029564	1.08E-09	2.94E-09
11.Erythromycin	0.40519606	3.34E-09	8.83E-09
04.Ertapenem	0.40213602	6.07E-09	1.56E-08
13.Chloramphenicol	0.32047385	1.14E-08	2.84E-08
01.Dibekacin	0.40081609	6.25E-07	1.52E-06
01.Arbekacin	0.06812612	2.22E-05	4.99E-05
04.Penicillin	0.16975191	0.00010596	0.00023259
06.Fosfomycin	0.20377845	0.00092122	0.00192814
01.Streptomycin	0.64436265	0.00177352	0.00362766
04.Cefixime	0.57753233	0.00379701	0.00742894
02.Spectinomycin	0.63133315	0.0045778	0.008766
01.Kanamycin	0.64968259	0.00658042	0.01220474
12.Tetracycline	0.66593795	0.00678041	0.01220474
12.Doxycycline	0.66593795	0.00678041	0.01220474
01.Paromomycin	0.6436223	0.01278714	0.02139722
01.Ribostamycin	0.6436223	0.01278714	0.02139722
01.Apramycin	0.21999053	0.01283833	0.02139722
01.Neomycin	0.64118168	0.01332109	0.02179814
01.Lividomycin	0.72433425	0.0820152	0.12100603
17.Colistin	0.27079314	0.27177517	0.38218383
22.Ethidium_Bromide	0.41192956	0.33828341	0.46129557
22.Chlorhexidine	0.41192956	0.33828341	0.46129557
13.Florfenicol	0.3683468	0.52588218	0.67613423
01.Isepamicin	0.9201573	0.68588507	0.85735634
16.Linezolid	0.46370341	0.72594412	0.8949996

Table S6. Distribution of isolates and observed antibiogram patterns in five major nosocomial pathogens under three S_{EV} thresholds (S_{EV} =100, ≥95, and ≥90).

Species	No. of isolates			No. of observed antibiogram patterns	
	Total	With AMRs (%)	Co-occurrence (%)[b]	Total	Co-occurrence (%)
S_{EV} = 100					
A. baumannii	10782	9840 (91.26)	1223 (12.43)	960	72 (7.50)
E. faecium	16235	15168 (93.43)	8311 (54.79)	532	87 (16.35)
K. pneumoniae	24396	21804 (89.38)	2244 (10.29)	5565	108 (1.94)
P. aeruginosa	13957	6726 (48.19)	2084 (30.98)	725	95 (13.10)
S. aureus	42016	35678 (84.92)	14782 (41.43)	1240	99 (7.98)
Total (nr)[a]	107386	89216 (83.08)	28644 (32.11)	9022 (8770)[a]	461 (209)[a]
$S_{EV} \geq 95$					
A. baumannii	10782	9929 (92.09)	514 (5.18)	1455	17 (1.17)
E. faecium	16235	16221 (99.91)	56 (0.35)	833	7 (0.84)
K. pneumoniae	24396	24393 (99.99)	479 (1.96)	5035	12 (0.24)
P. aeruginosa	13957	13943 (99.90)	24 (0.17)	1052	8 (0.76)
S. aureus	42016	37102 (88.30)	6345 (17.10)	1578	17 (1.08)
Total (nr)[a]	107386	101588 (94.60)	7418 (7.30)	9953 (9922)[a]	61 (30)[a]
$S_{EV} \geq 90$					
A. baumannii	10782	9934 (92.14)	505 (5.08)	1499	17 (1.13)
E. faecium	16235	16221 (99.91)	56 (0.35)	811	7 (0.86)
K. pneumoniae	24396	24393 (99.99)	444 (1.82)	5328	12 (0.23)
P. aeruginosa	13957	13950 (99.95)	21 (0.15)	1121	7 (0.62)
S. aureus	42016	37145 (88.41)	6449 (17.36)	1577	17 (1.08)
Total (nr)[a]	107386	101643 (94.65)	7475 (7.35)	10336 (10306)[a]	60 (30)[a]

[a] nr refers to nonredundant fingerprint patterns remaining after the removal of duplicate patterns, not nonredundant isolates.
[b] Co-occurrence percentages (%) are calculated using the number of AMR-positive isolates as the denominator.

SUPPLEMENTARY FIGURES

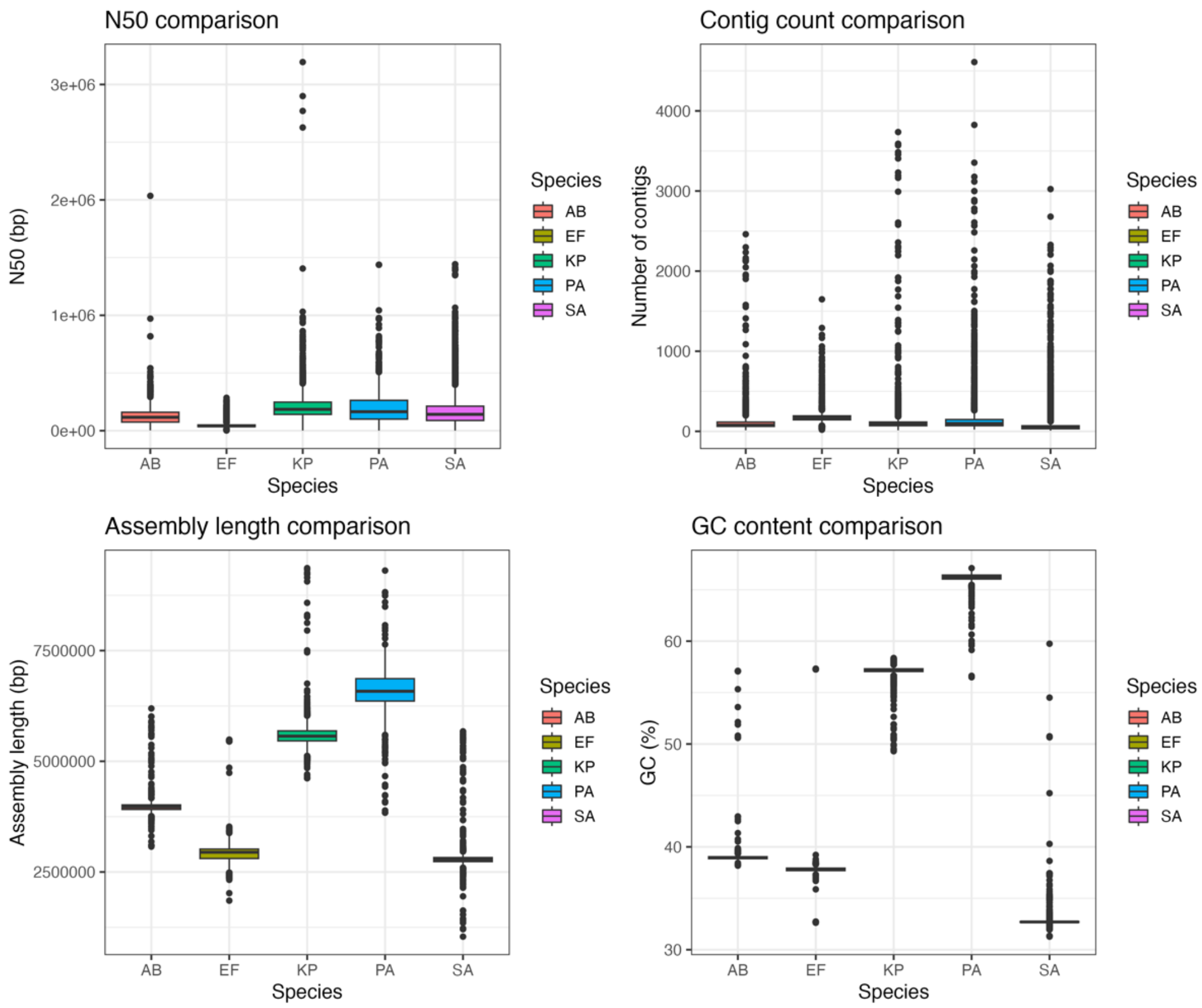

Figure S1. Comparative boxplot analysis of genome assembly quality metrics across five major nosocomial pathogens. The multi-panel boxplots summarize assembly quality statistics for de novo genome assemblies from *Acinetobacter baumannii* (AB), *Enterococcus faecium* (EF), *Klebsiella pneumoniae* (KP), *Pseudomonas aeruginosa* (PA), and *Staphylococcus aureus* (SA). The four panels display comparisons of N50 values, contig numbers, total assembly lengths, and GC contents across bacterial species. Boxplots represent the median, interquartile range (IQR), and distribution of assembly metrics for each species. Differences in assembly size and GC content were consistent with the known genomic characteristics of the corresponding bacterial species, while most assemblies exhibited favorable continuity metrics suitable for downstream antimicrobial resistance analyses. These metrics pertain to the original construction of the 5NosoAE dataset and were not newly generated as part of the present study.

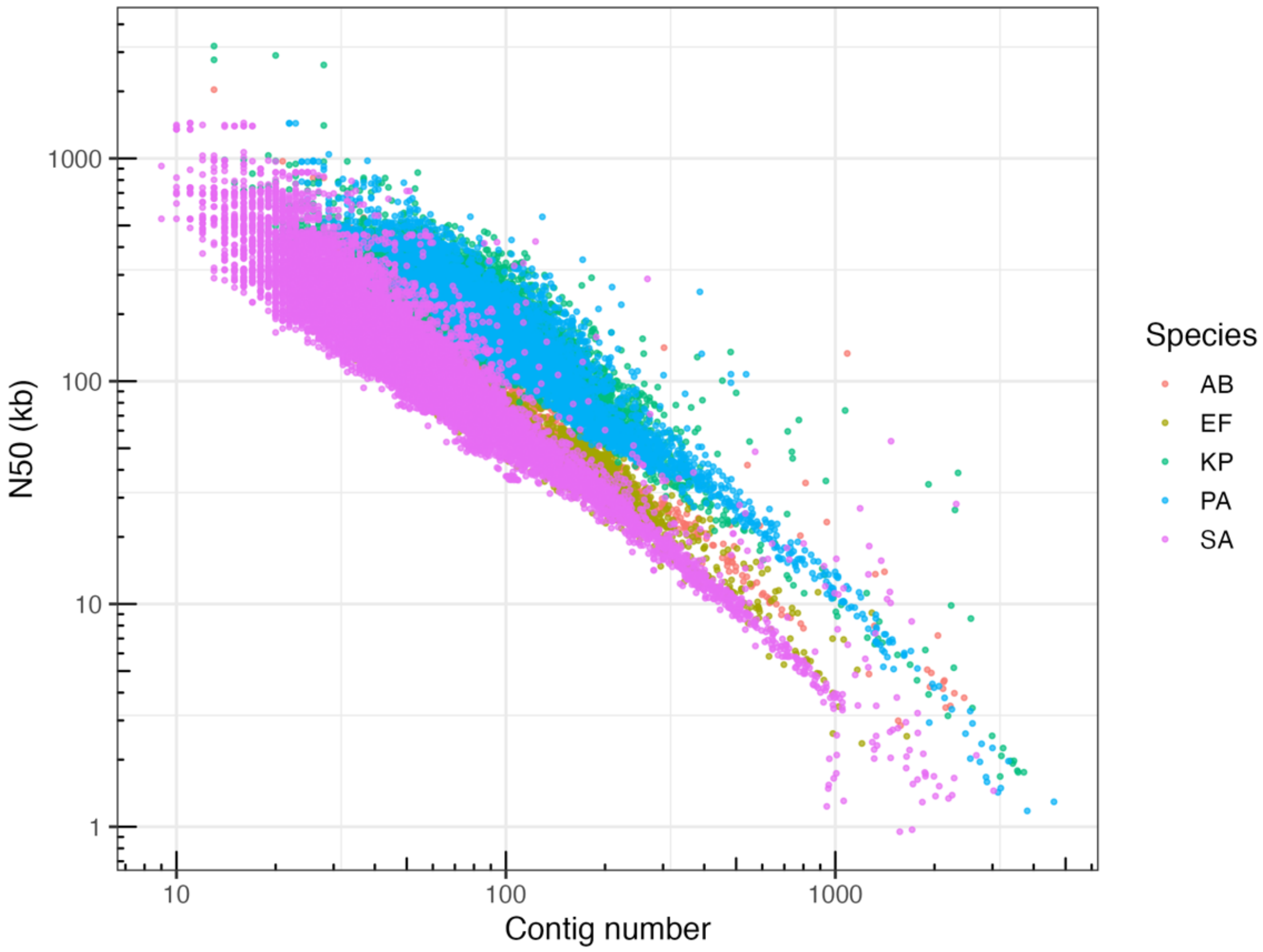

Figure S2. Scatter plot of assembly continuity metrics for de novo genome assemblies of five major nosocomial pathogens. The scatter plot illustrates the relationship between contig number and N50 value for 38,353 de novo genome assemblies from *Acinetobacter baumannii* (AB), *Enterococcus faecium* (EF), *Klebsiella pneumoniae* (KP), *Pseudomonas aeruginosa* (PA), and *Staphylococcus aureus* (SA). Both axes are displayed on a logarithmic scale. Each point represents an individual genome assembly, and colors indicate bacterial species. These metrics pertain to the original construction of the 5NosoAE dataset and were not newly generated as part of the present study.

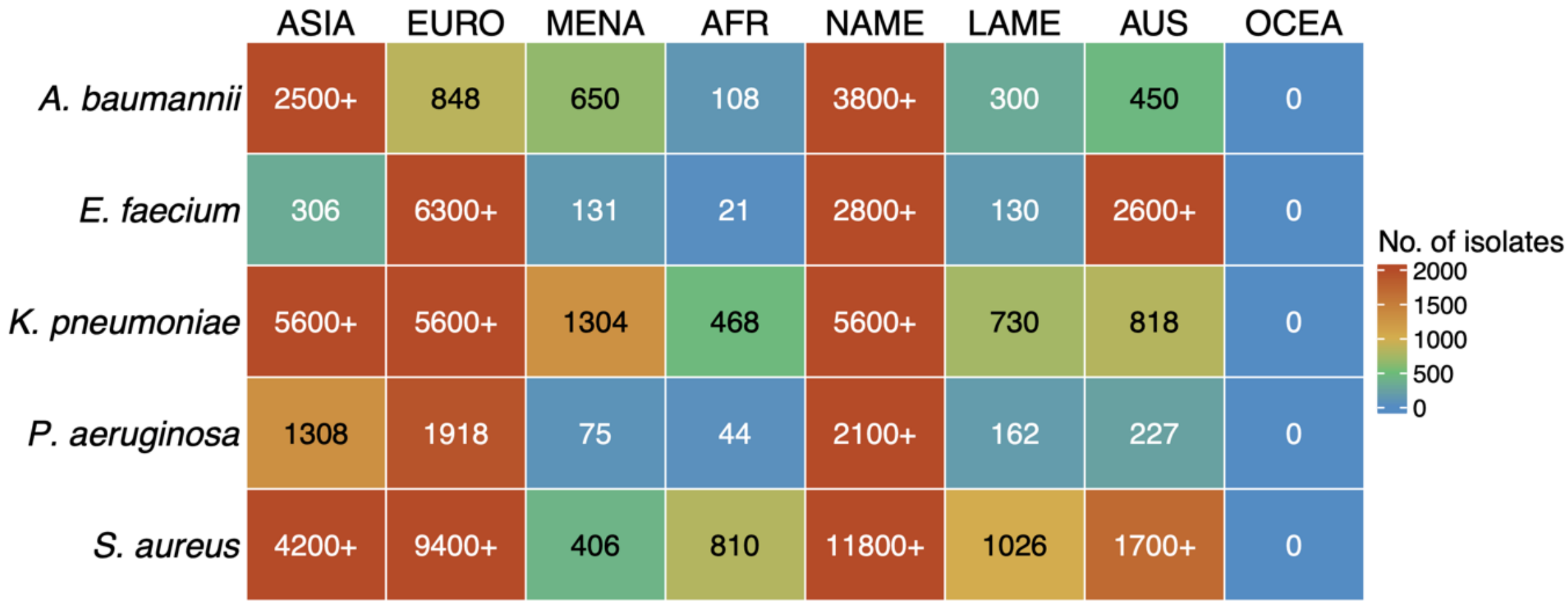

Figure S3. Heatmap of the regional distribution of the five major nosocomial pathogens. The values indicate the number of isolates collected in each geographic region; entries labeled with "+" denote lower-bound counts. Color intensity represents isolate abundance, which ranges from low, that is, blue; through green and yellow; to high, that is, red. Bacterial species names are written in italics. ASIA, Asia; EURO, Europe; MENA, Middle East and North Africa; AFR, Africa; NAME, North America; LAME, Latin America (excluding Mexico); AUS, Australia; OCEA, Oceania (excluding Australia).

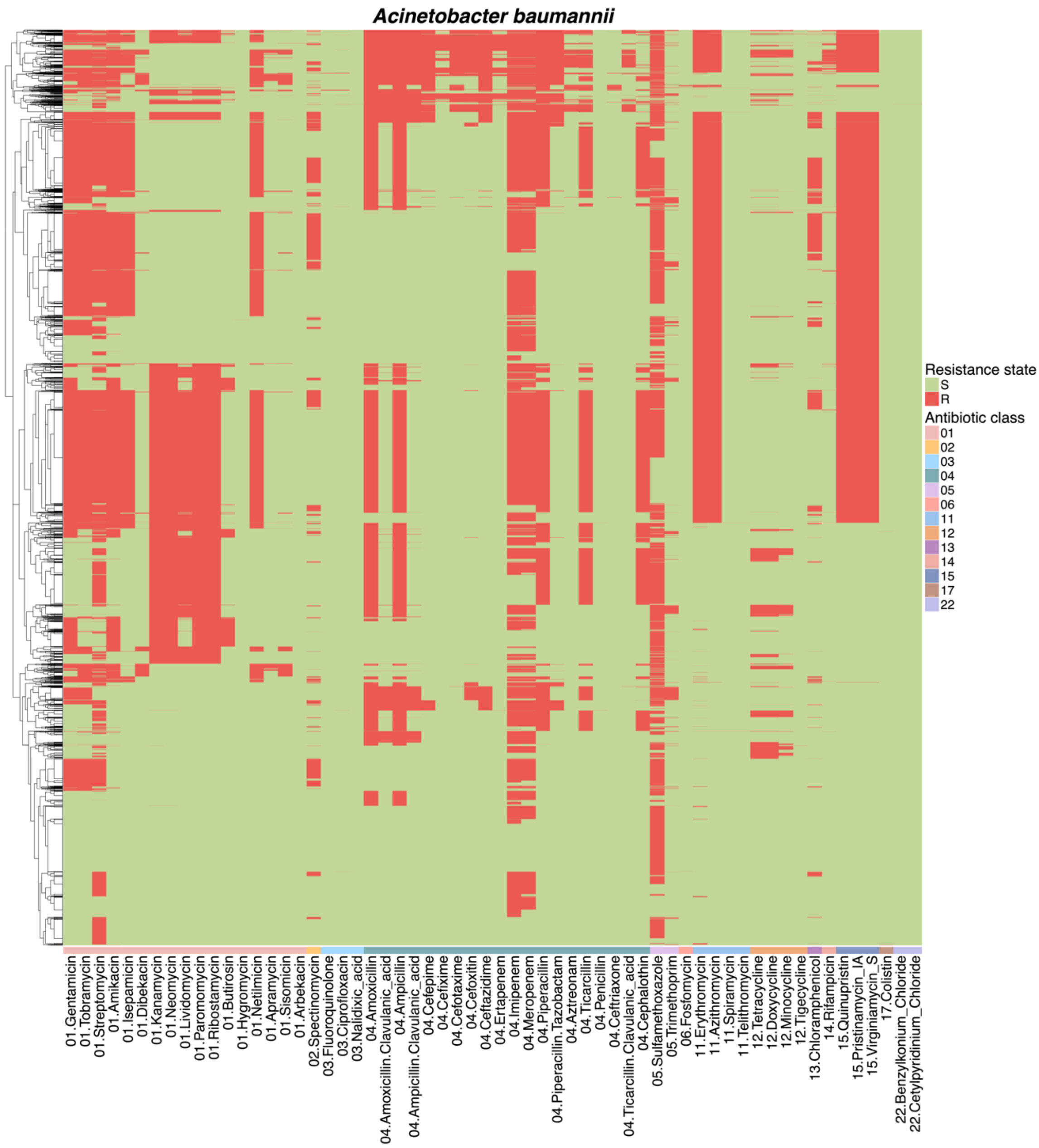

Figure S4. Antibiotic resistance profiles of *A. baumannii*.

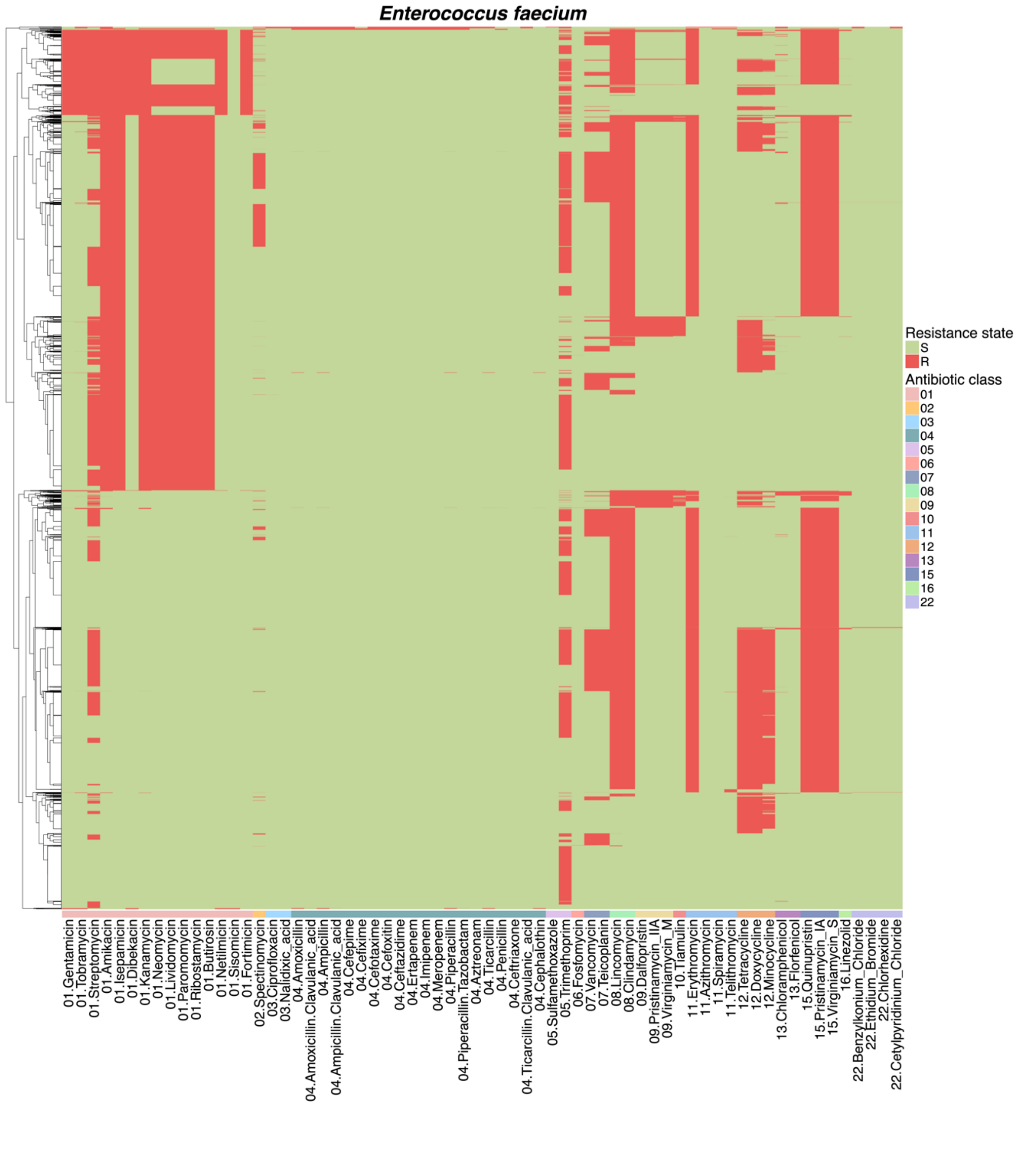

Figure S5. Antibiotic resistance profiles of *E. faecium*.

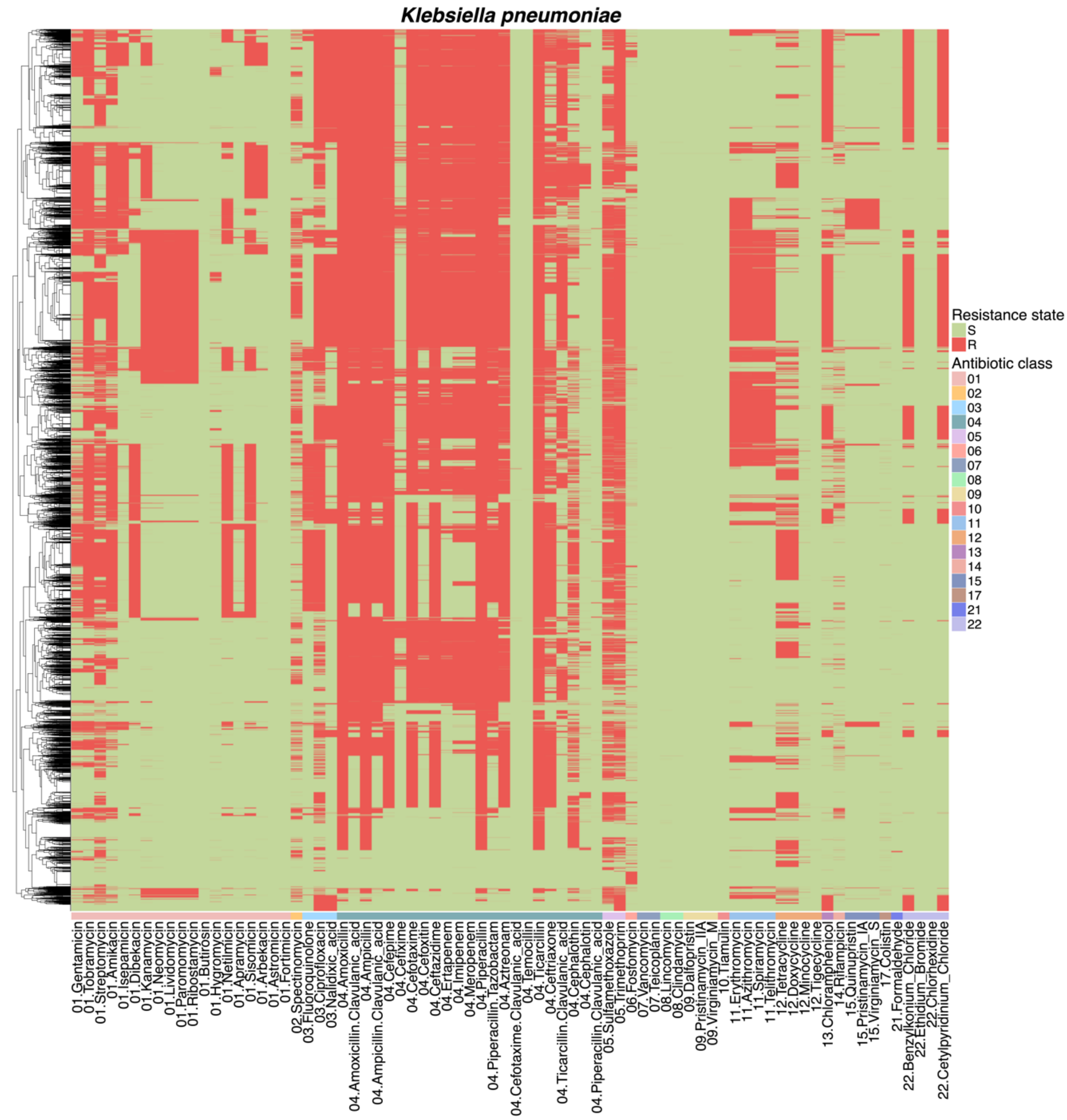

Figure S6. Antibiotic resistance profiles of *K. pneumonia*.

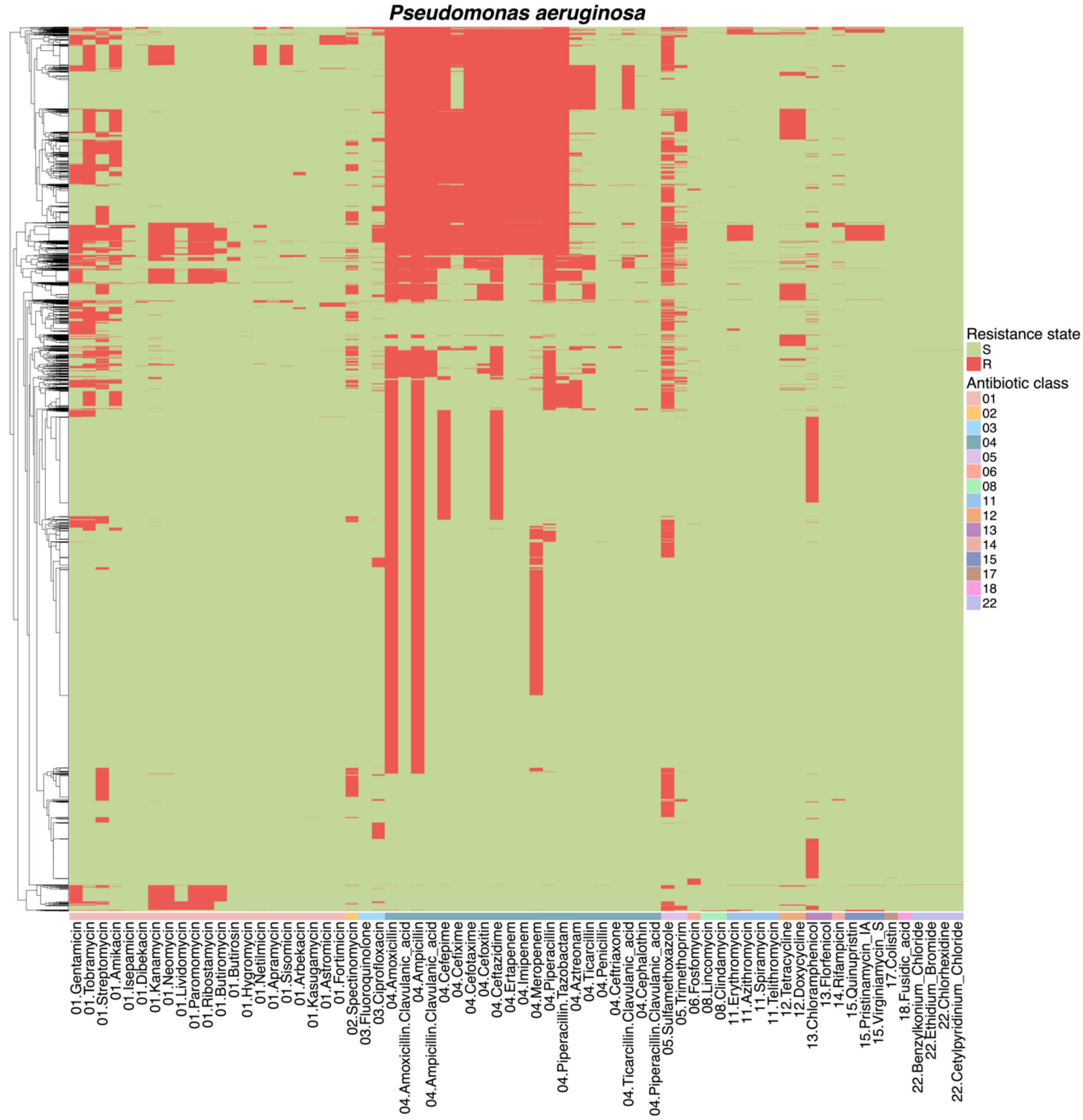

Figure S7. Antibiotic resistance profiles of *P. aeruginosa*.

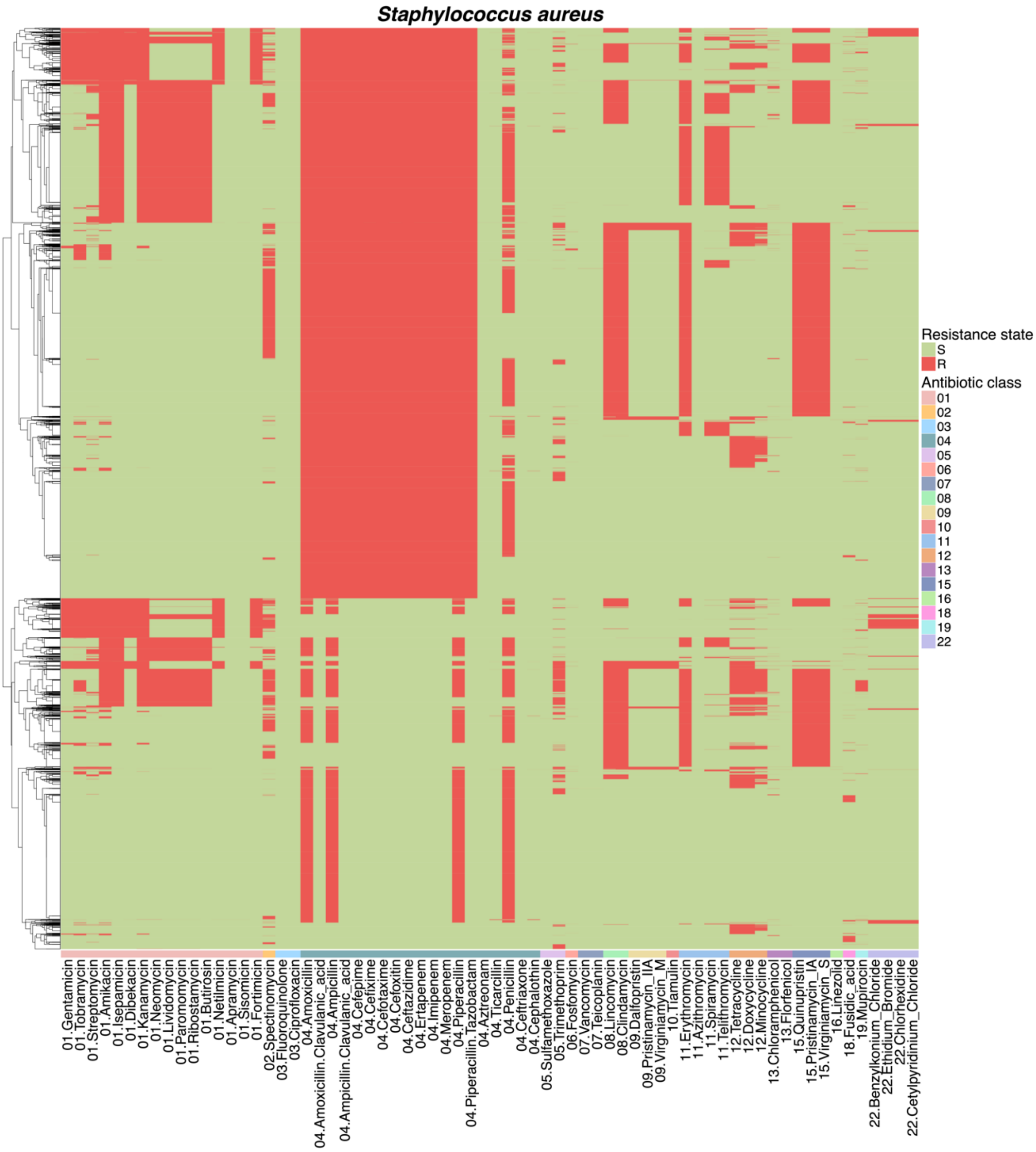

Figure S8. Antibiotic resistance profiles of *S. aureus*.

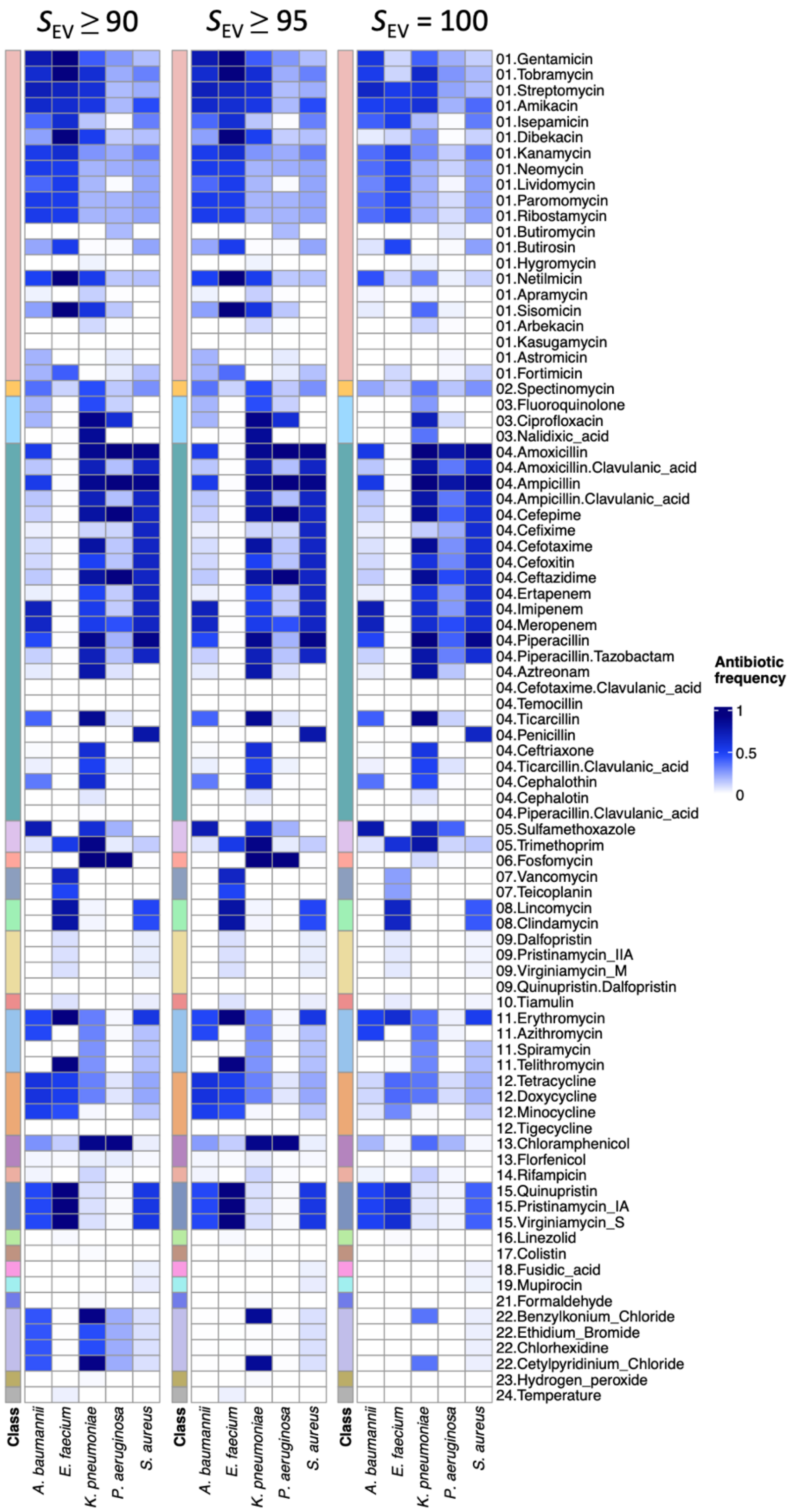

Figure S9. Heatmaps of antibiotic frequencies associated with resistance genes in the five major nosocomial pathogens at three S_{EV} thresholds (100, ≥95, and ≥90).

REFERENCES

1. Chen CC, Liu YY, Yang YC, Hsu CY. 2022. 5NosoAE: a web server for nosocomial bacterial antibiogram investigation and epidemiology survey. Nucleic Acids Res 50:W21-W28.

2. Sayers EW, Bolton EE, Brister JR, Canese K, Chan J, Comeau DC, Connor R, Funk K, Kelly C, Kim S, Madej T, Marchler-Bauer A, Lanczycki C, Lathrop S, Lu Z, Thibaud-Nissen F, Murphy T, Phan L, Skripchenko Y, Tse T, Wang J, Williams R, Trawick BW, Pruitt KD, Sherry ST. 2022. Database resources of the national center for biotechnology information. Nucleic Acids Res 50:D20-D26.

3. Chen S, Zhou Y, Chen Y, Gu J. 2018. fastp: an ultra-fast all-in-one FASTQ preprocessor. Bioinformatics 34:i884-i890.

4. Souvorov A, Agarwala R, Lipman DJ. 2018. SKESA: strategic k-mer extension for scrupulous assemblies. Genome Biol 19:153.

5. Gurevich A, Saveliev V, Vyahhi N, Tesler G. 2013. QUAST: quality assessment tool for genome assemblies. Bioinformatics 29:1072-5.

6. Bortolaia V, Kaas RS, Ruppe E, Roberts MC, Schwarz S, Cattoir V, Philippon A, Allesoe RL, Rebelo AR, Florensa AF, Fagelhauer L, Chakraborty T, Neumann B, Werner G, Bender JK, Stingl K, Nguyen M, Coppens J, Xavier BB, Malhotra-Kumar S, Westh H, Pinholt M, Anjum MF, Duggett NA, Kempf I, Nykasenoja S, Olkkola S, Wieczorek K, Amaro A, Clemente L, Mossong J, Losch S, Ragimbeau C, Lund O, Aarestrup FM. 2020. ResFinder 4.0 for predictions of phenotypes from genotypes. J Antimicrob Chemother 75:3491-3500.
